# Profiling the Oxylipin and Endocannabinoid Metabolome by UPLC-ESI-MS/MS in Human Plasma to Monitor Postprandial Inflammation

**DOI:** 10.1371/journal.pone.0132042

**Published:** 2015-07-17

**Authors:** Sandra Gouveia-Figueira, Jana Späth, Angela M. Zivkovic, Malin L. Nording

**Affiliations:** 1 Department of Chemistry, Umeå University, Umeå, Sweden; 2 Department of Nutrition, University of California Davis, Davis, United States of America; 3 Foods for Health Institute, University of California Davis, Davis, United States of America; University of East Anglia, UNITED KINGDOM

## Abstract

Bioactive lipids, including oxylipins, endocannabinoids, and related compounds may function as specific biochemical markers of certain aspects of inflammation. However, the postprandial responsiveness of these compounds is largely unknown; therefore, changes in the circulating oxylipin and endocannabinoid metabolome in response to a challenge meal were investigated at six occasions in a subject who freely modified her usual diet. The dietary change, and especially the challenge meal itself, represented a modification of precursor fatty acid status, with expectedly subtle effects on bioactive lipid levels. To detect even the slightest alteration, highly sensitive ultra-performance liquid chromatography (UPLC) coupled to electrospray ionization (ESI) tandem mass spectrometry (MS/MS) methods for bioactive lipid profiling was employed. A previously validated UPLC-ESI-MS/MS method for profiling the endocannabinoid metabolome was used, while validation of an UPLC-ESI-MS/MS method for oxylipin analysis was performed with acceptable outcomes for a majority of the parameters according to the US Food and Drug Administration guidelines for linearity (0.9938 < R^2^ < 0.9996), limit of detection (0.0005–2.1 pg on column), limit of quantification (0.0005–4.2 pg on column), inter- and intraday accuracy (85–115%) and precision (< 5%), recovery (40–109%) and stability (40–105%). Forty-seven of fifty-two bioactive lipids were detected in plasma samples at fasting and in the postprandial state (0.5, 1, and 3 hours after the meal). Multivariate analysis showed a significant shift of bioactive lipid profiles in the postprandial state due to inclusion of dairy products in the diet, which was in line with univariate analysis revealing seven compounds (NAGly, 9-HODE, 13-oxo-ODE, 9(10)-EpOME, 12(13)-EpOME, 20-HETE, and 11,12-DHET) that were significantly different between background diets in the postprandial state (but not at fasting). The only change in baseline levels at fasting was displayed by TXB_2_. Furthermore, postprandial responsiveness was detected for seven compounds (POEA, SEA, 9(10)-DiHOME, 12(13)-DiHOME, 13-oxo-ODE, 9-HODE, and 13-HODE). Hence, the data confirm that the UPLC-ESI-MS/MS method performance was sufficient to detect i) a shift, in the current case most notably in the postprandial bioactive lipid metabolome, caused by changes in diet and ii) responsiveness to a challenge meal for a subset of the oxylipin and endocannabinoid metabolome. To summarize, we have shown proof-of-concept of our UPLC-ESI-MS/MS bioactive lipid protocols for the purpose of monitoring subtle shifts, and thereby useful to address lipid-mediated postprandial inflammation.

## Introduction

The postprandial state, which is the state immediately following a meal, displays a dynamic course of events in response to the food ingested. It is an important period during which transient inflammation can occur. The current hypothesis is that during the normal homeostatic postprandial response, inflammatory compounds are transiently produced, which cause no harmful effects. In the case of a maladaptive postprandial response, during the so-called “postprandial metabolic inflammation”, an exaggerated and protracted inflammation may occur, linked to an unfavorable immune response [1]. This postprandial modification of innate immunity has been suggested to be responsible for the adverse effects of certain dietary fatty acids on, for instance, cardiovascular health. In general, the ω6 polyunsaturated fatty acids (PUFA) promote postprandial inflammation, while the ω3 PUFA suppress it. But the effects of fatty acid meal composition on inflammatory events and cardiovascular health are conflicting and have mainly been studied in models of chronic exposures, whereas the significance of postprandial adaptation remains unclear [2–7]. This gap in knowledge highlights the need for reliable biochemical markers of lipid-mediated postprandial inflammation. Several candidates have been investigated, including cytokines and bioactive lipids [8–11]. The bioactive lipid family of oxylipins is a particularly compelling group of molecules for postprandial studies due to its role in endothelial inflammation and cardiovascular disease [12, 13].

The term oxylipin includes ω6 derived 20-carbon eicosanoids (e.g. prostaglandins and leukotrienes), as well as other oxidized fatty acid metabolites ranging from well-established inflammatory mediators to potential key molecules of inflammation [14]. Oxylipins have long been recognized for their involvement in inflammation through the catabolic pathways of cyclooxygenase (COX), lipooxygenase (LOX), and cytochrome P450 (CYP). Furthermore, accumulating evidences suggest that another family of bioactive lipids, the endocannabinoids, share the same catabolic pathways and may be responsible for at least some of the beneficial anti-inflammatory effects from drugs inhibiting these enzymes [15]. Endocannabinoids are also fatty acid metabolites with signaling properties, but unlike oxylipins, they are able to bind to and activate cannabinoid receptors [16]. Most studied are anandamide (AEA) and 2-arachidonyl glycerol (2-AG), which are associated, among other things, with the regulation of inflammation and appetite [15, 17–19]. Other *N*-acylethanolamines (NAEs), monoacylglycerols (MAGs) and related compounds are characterized as “cannabimimetic” when they are able to activate cannabinoid receptors, or “entourage compounds” which are unable to activate CB receptors, but still modify the activity of true endocannabinoids by inhibiting their degradation and metabolism [20–23].

Even though oxylipins and endocannabinoids are connected, they are rarely investigated together. One reason for this lack of experiments is the challenging analytical protocol. Nevertheless, simultaneous analysis of oxylipins and endocannabinoids has occasionally been performed, revealing valuable information such as changes associated with dietary fish oil in mice [24–27]. Furthermore, separate investigations of oxylipins and endocannabinoids have underscored the importance of studies on the effect of dietary fatty acids on circulating bioactive lipid levels [28–30]. However, the postprandial responsiveness *per se* of oxylipins and endocannabinoids is still poorly understood, which is necessary to elucidate if these compounds are to be used as markers for studies on postprandial inflammation. The challenge meal study design offers a suitable approach to test if a method is able to detect subtle shifts in the metabolome, as illustrated by anandamide and other NAE shifts in the postprandial state [31, 32]. Furthermore, the feasibility of a challenge meal has previously been demonstrated by the reproducibility of individual metabolic responsiveness of circulating fatty acids [33]. Therefore, the challenge meal study design was used to test the performance of ultra-performance liquid chromatography (UPLC) coupled with electrospray ionization (ESI) tandem mass spectrometry (MS/MS) methods for the analysis of a selected panel of oxylipins, endocannabinoids, cannabimimetic and entourage compounds. Thereby, we did not relate the levels to free fatty acids (like in [31]), but we extended the investigation of the postprandial state compared to [32], and added compounds beyond what has been reported previously [31–33].

The endocannabinoid metabolome was analyzed using a previously validated UPLC-ESI-MS/MS method [34], while a novel UPLC-ESI-MS/MS method for analysis of the oxylipin metabolome was developed and validated for application to human plasma at fasting and in the postprandial state after a well-defined meal. The measurements were repeated for the same individual on two different diets, vegan and vegetarian, to investigate the effects of modifications of precursor fatty acid status introduced both by the background diet, and the challenge meal itself, on the circulating bioactive lipid levels. Such subtle shifts in phenotype and responsiveness are difficult to detect with markers that were developed to detect instead the major changes that occur in disease vs. health. For example, C reactive protein (CRP), the most well-known marker of systemic inflammation, differentiates between individuals with overt disease compared with healthy controls [35, 36], and changes in response to major shifts in diet [37, 38]. However, CRP does not always change in response to more subtle shifts in diet or weight loss [39, 40], and does not change in the postprandial state [41–44]. It is increasingly recognized that quantification of a standardized perturbation of metabolic homeostasis induced by dietary interventions is more informative than quantification of only the homeostatic situation (at fasting) [45]. Thus, more sensitive and specific markers of inflammation that can detect more subtle differences between different phenotypes and shifts in response to meals and changes in diet are needed.

To that end, the aim of this study was to develop comprehensive methods to analyze the circulating oxylipin and endocannabinoid metabolome (totaling fifty-two metabolites) with sufficient sensitivity to detect and quantify subtle differences introduced by changes in background diet before and after a challenge meal at multiple test occasions. We applied our validated method for endocanabinoids and related compounds [34], and validated a new method for oxylipins Despite the fact that several methods dealing with quantification of oxylipins previously have been reported [24, 33, 46–49], method validation when using new equipment is necessary to ensure data quality and reproducibility [24]. Furthermore, there is a great need to employ methods with improved sensitivity and specificity due to the extreme low levels that most of the bioactive lipids are found at in healthy individuals.

By studying the postprandial variability of thirty-five circulating oxylipins, and twelve endocannabinoids and similar compounds, we were able to show proof-of-concept of our methods for the purpose of monitoring lipid-mediated postprandial inflammation. Our results highlighted that a well-defined challenge meal may reduce noise in the postprandial bioactive lipid metabolome compared to the fasting state, and thereby more differences in relation to subtle effects (here represented by changes in background diet) may be detected in the postprandial state than at fasting.

## Materials and Methods

### Study design

This was a pilot study using the challenge meal study design of a single female subject continuously eating the diet of her choice, who freely changed diet from vegan to vegetarian. From this point the vegan diet will be denominated as “usual diet” and vegetarian diet as “modified diet” since we are not making any attempt to relate the actual changes in diet to biological markers, neither on individual level nor on population level. The subject was not fed anything or told to eat anything specific as part of this study but instead was a free-living individual. She was a healthy female with no disease diagnoses, except for celiac disease, with a body mass index of 20, 29 years of age when the study began, and 30 when it ended. Body weight was monitored throughout the study and fluctuated between 55.5 to 57 kg. She was tested three times on each background diet using a challenge meal of bananas (in agreement with her dietary choice according to her diet records). The study protocol was defined according to the human research ethics in the Declaration of Helsinki and did not require anything beyond that the subject was able to donate blood safely since she was recruited under a methods development study protocol, which was approved by the Institutional Review Board of the University of California, Davis. Written informed consent was obtained prior to study commencement.

After being on her usual diet for 5 years, refraining from meat and dairy products, the subject modified her diet by inclusion of dairy products. Other consumed food products, including abstaining from eating all gluten containing foods, remained similar as (assessed by comparison of seven-day dietary records from both dietary phases). The first set of meal challenges was performed during a period of eight days before inclusion of dairy products to the background diet. The amount of dairy products was then gradually increased during one year to a steady state, which was kept for 33 weeks until the second set of meal challenges was performed.

Dietary intake was assessed with seven-day dietary records. Nutrient information for each day was analyzed from the dietary records using the NutriHand program (Nutrihand Inc., Soraya, CA) from reference nutrient data for individual foods using the USDA National Nutrient Database for Standard Reference. Nutrient data were averaged together for all 7 days within each dietary period and then compared by two-tailed *t*-tests. The main weekly differences between the diets during the 33 weeks were on average: 2 L yoghurt, 1.7 eggs, 200 g cheese and 2.1 dL cream and butter per week in the modified diet. This change did not affect the overall consumption of calories, proteins, total fats or carbohydrates, but resulted in a shift in the relative composition of fatty acids (**S1 Table**). More specifically, during the modified diet the subject consumed lower amounts of fiber, magnesium, copper, vitamin C, total PUFAs and 18:2n6, and higher amounts of calcium, vitamin B12, total saturated fatty acids (as well as individual short and medium chain fatty acids), the monounsaturated fatty acid 16:1 and cholesterol.

**Fig 1:**
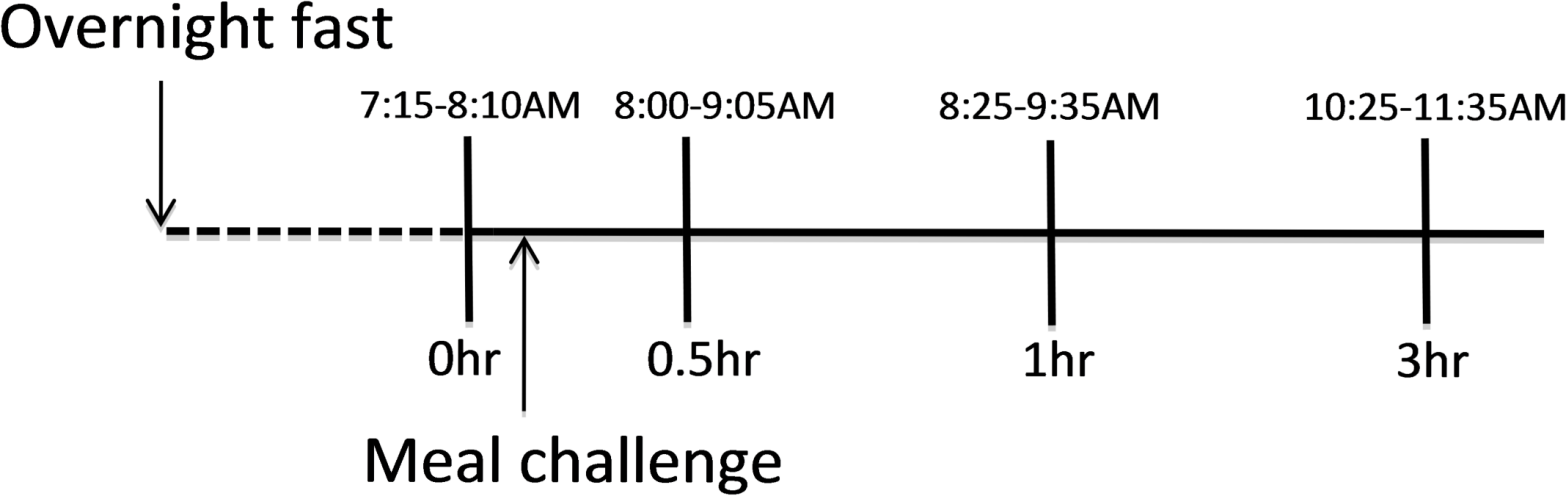
The study design. After an overnight fast, blood was collected and thereafter the challenge meal was consumed. Postprandial blood collection was done at 0.5, 1, and 3 h after finished meal. The meal challenge was repeated at six occasions in total. Hour of blood collection varied between experiments, illustrated by time spans.

The subject was tested three times on each background diet on three non-consecutive days during a time frame of up to two weeks totaling six test occasions. No major lifestyle changes occurred during the course of the study, as monitored with physical activity and health questionnaires. On each test day, the subject came to the study center after an overnight fast, and was weighed and underwent a questionnaire to exclude recent changes in diet, lifestyle, physical activity, medication or supplement use. The challenge meal was then consumed within 5–7 min and consisted of 335.6 g–348.5 g of banana providing approximately 15% of daily calories. A fasting blood sample was collected and postprandial blood draws were performed at 0.5, 1, and 3 hours after the finished meal (**Fig 1**) using lavender-top EDTA tubes. Whole blood was centrifuged in a tabletop ultracentrifuge for 10 minutes at room temperature at 3000 rpm within one hour of collection. Plasma (containing both unesterified and esterified fatty acids) was then collected and aliquoted and stored at –80°C until analysis.

### Chemicals and reagents

Despite the excessive number of potential oxylipins, only 50 are usually reported in human plasma [27, 47]. Gathering that information and based on the purpose of our research topic, we selected 37 oxylipins for inclusion in our method to profile the oxylipin metabolome. Furthermore, 15 NAEs, MAGs and related compounds were analyzed in order to profile the endocannabinoid metabolome. Chemical structures of all analytes are shown in **S1 Fig.**

The following native, internal, and recovery standards were purchased from Cayman Chemicals (Ann Arbor, MI, USA): AEA, 2-AG, *O*-AEA, 2-AGe, NADA, PEA, OEA, DEA, NAGly, EPEA, DHEA, POEA, LEA, SEA, 2-LG, AEA-d_8_, 2-AG-d_8_, OEA-d_4_, and DHEA-d_4_ (for analysis of NAEs, MAGs and related compounds); and PGF_2α_, PGE_2_, TXB_2_, PGD2, 5(6)-EET, 8(9)-EET, 11(12)-EET, 14(15)-EET, 5,6-DHET, 8,9-DHET, 11,12-DHET, 14,15-DHET, 9(10)-EpOME, 12(13)-EpOME, 9(10)-DiHOME, 12(13)-DiHOME, 5-HETE, 8-HETE, 9-HETE, 11-HETE, 12-HETE, 15-HETE, 20-HETE, 9-HODE, 13-HODE, 15(S)-HETrE, 12-HEPE, 17-HDoHE, 5-oxo-ETE, 12-oxo-ETE, 15-oxo-ETE, 13-oxo-ODE, LTB4, Resolvin D2, Resolvin D1, 12-[[(cyclohexylamino)carbonyl]amino]-dodecanoic acid (CUDA), 12(13)-DiHOME-d_4_, 12(13)-EpOME-d_4_, 9-HODE-d_4_, PGE_2_-d_4_, and TXB_2_-d_4_ (for oxylipin analysis). The oxylipins 9,10,13-TriHOME and 9,12,13-TriHOME were obtained from Larodan (Sweden, Malmö). Acetonitrile (ACN) and methanol (MeOH) were from Merck (Darmstadt, Germany). Isopropanol was from VWR PROLABO (Fontenay-sous-Bois, France). Ammonium acetate (CH_3_COONH_4_) was purchased from Scharlau Chemie (Barcelona, Spain). Acetic acid was purchased from Aldrich Chemical Company, Inc. (Milwaukee, WI, USA). Butylhydroxytoluene (BHT) was from Cayman Chemical (Ann Arbor, MI, USA) and ethylenediaminetetraacetic acid (EDTA) from Fluka Analytical, Sigma-Aldrich (Buchs, Switzerland). Glycerol was from Fischer Scientific (Loughborough, UK). All solvents and chemicals were of HPLC grade or higher. Water was purified by a Milli-Q Gradient system (Millipore, Milford, MA, USA).

#### Internal standards (IS)

Deuterated compounds were used as internal standards (IS) for quantification purposes and were added to samples before extraction to mimic the extraction of the endogenous compounds. Three IS were used for endocannabinoid quantification (AEA-d_4_, OEA-d_4_ and 2-AG-d_8_), and five for oxylipin quantification (12(13)-DiHOME-d_4_, 12(13)-EpOME-d_4_, 9-HODE-d_4_, PGE_2_-d_4_, and TXB_2_-d_4_). For each native compound, a suitable IS was selected based on structural similarities (**S2 Table**). Recovery rates of each IS were calculated by adding the recovery standard DHEA-d_4_ (endocannabinoids) or CUDA (oxylipins) in the last step before analysis [46].

#### Standard stock solutions

Analytical quantification standards were used as ready-made standard stock solutions or as solutions prepared from solid substances and stored at -80 °C. 2-AG, *O*-AEA, 2-AGe, 2-LG and 5(6)-EET were prepared and stored in ACN and the other standards were prepared and stored in ethanol to yield a final stock solution concentration of 250 μg/mL (endocannabinoids) and 100 or 1000 μg/mL (oxylipins). Stock solutions of IS were prepared to reach a final concentration of 40 μg/mL (endocannabinoids) and 10 μg/mL (oxylipins).

#### Standard curve preparation

Further dilutions of each stock solution were made with methanol at ten different calibration levels for quantification purposes, prepared fresh on a weekly basis for endocannabinoids and stored at -80°C for oxylipins (**S3 and S4 Tables**). The lowest concentration in each calibration curve was further diluted to determine limit of quantification (LOQ) and detection (LOD) at a signal to noise ratio (S/N) of 10 and 3, respectively.

### Endocannabinoid quantification

Analysis of NAEs, MAGs and related compounds was done according to our previously validated method [34]. Briefly, the plasma samples (300–500 μL) were subjected to solid phase extraction (SPE) using Waters Oasis HLB cartridges (60 mg of sorbent, 30 μm particle size). A MiniVac system (Farmingdale, NY, USA) was used to evaporate the eluates from the SPE cartridges. The analytes were then reconstituted in 100 μL MeOH and 10 μL recovery standard was added before UPLC-ESI-MS/MS analysis. The UPLC system consisted of a Waters Acquity Ultra Performance equipment (Milford, MA, USA) with a binary pump, a thermostated column compartment and an autosampler. LC separation was achieved using a Waters BEH C_18_ column (2.1 mm x 150 mm, 1.7 μm particle size) at 60°C, with an injection volume of 10 μL. MilliQ water (**A**) and 10 mM CH_3_COONH_4_ in MeOH (**B**) were used as mobile phase following this gradient elution: 0.0–9.0 min 79% **B**, 9.0–9.5 min 79–90% **B**, 9.5–10.5 min 90% **B**, 10.5–14.0 min 79% **B**, at a flow rate of 0.4 mL/min. The autosampler temperature was maintained at 10°C. The mass analysis was done on a Waters triple quadrupole MS (Micromass Quattro Ultima) equipped with an electrospray ionization source operating in positive mode (ESI^+^). N_2_ was used as drying gas (60 L/hr) and Ar as nebulization gas (650 L/hr). Source and desolvation temperatures were 150°C and 350°C, respectively.

### Oxylipin quantification

A previously published SPE protocol was adapted for extraction of oxylipins from plasma [46]. On the day of extraction, the plasma samples were thawed at room temperature and centrifuged. SPE Waters Oasis HLB cartridges (60 mg sorbent, 30 μm particle size) were first washed with 2 mL of ethyl acetate, 4 mL of methanol, and 4 mL of 95:5 v/v water/MeOH with 0.1% acetic acid (WS). A quantitative volume of plasma (300–500 μL depending on the amount supplied) was loaded onto the SPE cartridges and spiked with 10 μL of internal standard solution (50 ng/mL for 12(13)-DiHOME-d_4_ and 12(13)-EPOME-d_4_, and 25 ng/mL for 9(S)-HODE-d_4_, PGE_2_-d_4_ and TXB_2_-d_4_) and 10 μL antioxidant solution (0.2 mg/mL BHT/EDTA in methanol/water (1:1)). The SPE cartridges were then washed with 4 mL of WS, dried under high vacuum for about 1 minute, and eluted with 2 mL methanol and 2 mL ethyl acetate into polypropylene tubes containing 6 μL of a glycerol solution (30% in methanol). Glycerol operates as a trap solution for the analytes. Eluates were evaporated under vacuum (MiniVac system, Farmingdale, NY, USA) and residues were then reconstituted in 100 μL methanol and tubes were vortexed. The solutions were transferred to LC vials and 10 μL of the recovery standard solution was added (5 ng/mL CUDA) and UPLC-AJST-ESI-MS/MS analysis was performed immediately in randomized order. The Agilent UPLC system (Infinity 1290) was coupled to an Agilent 6490 Triple Quadrupole system equipped with the iFunnel Technology source (Agilent Technologies, Santa Clara, CA, USA). The UPLC column used was the same as described for the endocannabinoid methodology, a Waters BEH C_18_ column (2.1 mm x 150 mm, 2.5 μm particle size), and 10 μL injection volume was employed. Different mobile phase composition and different gradients were compared in order to achieve optimal separation for all compounds, especially between critical isomer pairs. The optimal conditions were found with 0.1% acetic acid in MilliQ water (**A**) and acetonitrile:isopropanol (90:10) (**B**) using the following gradient: 0.0–3.5 min 10–35% **B**, 3.5–5.5 min 40% **B**, 5.5–7.0 min 42%B, 7.0–9.0 min 50% **B**, 9.0–15.0 min 65% **B**, 15.0–17.0 min 75% **B**, 17.0–18.5 min 85% **B**, 18.5–19.5 min 95% **B**, 19.5–21 min 10% **B**, 21.0–25.0 min 10% **B** with constant flow rate of 0.3 mL/min. The autosampler temperature was kept at 10°C and the column at 40°C. The mass analysis was done in negative mode (AJST-ESI^-^). For each oxylipin compound, precursor ions [M-H]^-^, product ions and optimal collision energies were established for each MRM transition using the MassHunter Optimizer software. The Source and iFunnel Optimizer was executed for optimizing seven parameters (capillary, nozzle and nebulizer voltage, gas temperature and flow, and sheath gas temperature and flow). A compromise had to be made between the optimal conditions for each compound and the final AJST-ESI^-^ conditions were: capillary and nozzle voltage at 4000 V and 1500 V, respectively, drying gas temperature 230°C with a gas flow of 15 L/min, sheet gas temperature 400°C with a gas flow of 11 L/min, the nebulizer gas flow was 35 psi, and iFunnel high and low pressure RF at 90 and 60 V, respectively. The dynamic MRM option was used and performed for all compounds with optimized transitions and collision energies. Integration of all peaks was manually performed using the MassHunter Workstation software.

### Oxylipin method validation

The method was validated according to the US Food and Drug Administration (FDA) guidelines over three consecutive days for linearity, LOD, LOQ, inter- and intraday accuracy and precision, recovery and stability [50], in line with our previously validated method for endocannabinoid analysis [34]. However, we used the LOD and LOQ definition based on the signal to noise response, which not automatically translates to the lower limit of quantification (LLOQ) corresponding to the lowest point in the calibration curve used in the FDA guidelines.

#### Linearity

At least fourteen different concentrations of each standard were prepared and analyzed in triplicate. Calibration curves were calculated by the least-squares linear regression method using the equation y = m(x)+b, where “y” is equal to the response ratios (native standard peak area/internal standard peak area), “m” is equal to the slope of the calibration curve, “x” is equal to the on column concentration of the native analyte and “b” is equal to the y-interception of the calibration curve. Equal weighting factor was used. For concentration determinations of each analyte in real samples, a 10-point calibration curve was used (**S4 Table**). The peak area ratio between the analyte and its corresponding internal standard (**S2 Table**) were used (“y”) and the concentrations (“x”) were calculated using the calibration curve equation.

#### Limit of detection (LOD) and Limit of Quantification (LOQ)

The limit of detection (LOD) was defined as the concentration that resulted in a peak with a signal-to-noise ratio (S/N) greater than three and the limit of quantitation (LOQ) was defined as S/N greater than 10.

#### Accuracy and precision

Accuracy and precision were determined using 4 quality control (QC) samples of 750 μL 100 mM phosphate buffer saline (PBS) spiked with native standard mixtures at different concentrations (corresponding to on-column values of 96; 24; 12 and 2.9 pg/μL). On three non-consecutive days, three replicates of each QC sample were extracted by SPE as described above for plasma samples and analyzed by UPLC-AJST-ESI-MS/MS together with a complete set of calibration standards. Calibration curves obtained for each batch were used to determine the QC sample concentration.

The intraday accuracy was determined as the percent difference between the expected concentration and the mean concentration for each analytical run (n = 3). The interday accuracy was determined as the percent difference between the expected concentration and the mean concentration on the three different days (n = 9). The acceptable range of inter- and intraday accuracy was considered to be 80–120%.

The coefficient of variation (% CV) for the mean concentration was used to calculate the intra- and interday precision (n = 3 and n = 9, respectively). A CV less than 20% was considered acceptable.

#### Recovery

Internal standard recovery rates were established for different matrices in triplicates by adding a recovery standard (CUDA) immediately before UPLC-MS/MS injection to account for changes in volume and instrument variability. Accordingly, the internal standard recovery was calculated by spiking, in triplicates, 750 μL of PBS (100 mM) with 10 μL of internal standard solutions at four different levels (corresponding to on-column values of 9.1, 4.5, 2.3 and 1.1 pg/μL) prior to SPE extraction. Matrix-dependent recovery was established by spiking 10 μL internal standards in a similar manner to human plasma). A 5 point calibration curve was generated by plotting each internal standard on column concentration vs IS/CUDA area to calculate the amount of each internal standard recovered through all the extraction steps, expressed as the percentage of the expected value. This procedure was applied to all the samples analyzed.

#### Stability

The stability of each analyte was determined by measuring sample concentrations immediately after collection and at different time points after storage at -20 and -80°C, and after two freeze-thaw cycles. Stability of analytes in working solutions was determined by comparing fresh solutions and solutions stored at -80°C for four weeks.

### Statistical analysis

Metabolite levels were calculated and expressed as mean ± SEM, using GraphPad Prism 6 (San Diego, CA, U.S.A.). To detect significant temporal changes in the oxylipin and endocannabinoid metabolome during the postprandial response, differences between compound concentrations across the time points were assessed using one-way ANOVA with post hoc Tukey’s multiple comparison test at α = 0.05 considered significant. Two-tailed and paired Student’s *t*-test corrected for multiple comparison with the Holm-Sidak method (α = 0.05) was used to detect significant differences of each metabolite concentration at each time point between the vegan and vegetarian background diet. Multivariate data analysis was done using SIMCA software (Version 13.0, Umetrics, Umeå, Sweden). All data was mean centered and scaled to unit variance before modeling.

## Results

### Endocannabinoid quantification

In the 24 samples analyzed, a total of 12 NAEs, MAGs, and related compounds were detected with average levels ranging from 0.1 to 211 nM (**Table 1**). POEA was found at the highest levels followed by 2-LG, 2-AG, SEA and LEA. All 15 compounds analyzed were present in all samples, except for 2-AG and 2-LG (detected in all but one), EPEA (only detected in 3 vegan samples) and 2-AGe and NADA, which were not detected at all (**S5 Table**).

**Table 1:** Average levels (nM) ± SEM of *N*-acylethanolamines (NAEs), monoacylglycerols (MAGs) and related compounds in the endocannabinoid metabolome of human plasma at fasting (baseline) and in the fasting and postprandial state (all samples) in a subject on usual (vegan) or modified (vegetarian) diet.

				Usual Diet			Modified Diet		
Compound	Abbreviation	Fatty acid precursor	Chemical class	Baseline (n = 3)	All samples (n = 12)	Range	Baseline (n = 3)	All samples (n = 12)	Range
Arachidonoyl glycine	NAGly	Arachidonic acid (20:4n6)	*N*-acylglycine	1.69±0.36	1.58±0.18	0.68–3.15	1.17±0.18	1.09±0.20	0.64–3.20
Eicosapentaenoyl ethanolamide	EPEA	Eicosapentaenoic acid (20:5n3)	NAE	0.109±0.01	0.30±0.05	0.1–0.48	*ND*	*ND*	*ND*
Palmitoleoyl ethanolamide	POEA	Palmitoleic acid (16:1n7)	NAE	89.300B114.84	70.0±6.9	46.1–118	140.5±56	87.5±18.1	22–210
Docosahexaenoyl ethanolamide	DHEA	Docosahexaenoic acid (22:6n3)	NAE	1.41±0.44	1.24±0.16	0.73–2.28	1.19±0.17	1.26±0.09	0.89–2.09
Arachidonoyl ethanolamide	AEA	Arachidonic acid (20:4n6)	NAE	1.74±0.32	1.44±0.17	0.91–2.81	1.24±0.32	1.21±0.10	0.82–1.86
Linoleoyl ethanolamide	LEA	Linoleic acid (18:2n6)	NAE	9.01±0.39	7.84±0.44	5.47–10.2	8.65±0.77	8.99±0.86	4.05–15.9
2-Arachidonoyl glycerol	2-AG	Arachidonic acid (20:4n6)	MAG	29.2±7.05	24.9±5.24	0.81–50.9	17.7±9.7	16.9±2.6	7.23–37.2
2-Linoleoyl glycerol	2-LG	Linoleic acid (18:2n6)	MAG	40.2±12.72	44.1±7.6	0–96.66	42.8±10.2	38.6±3.4	20.7–62.6
Palmitoyl ethanolamide	PEA	Palmitic acid (16:0)	NAE	1.90±0.34	1.60±0.15	1.07–2.64	1.60±0.30	1.58±0.16	0.98–2.89
Docosatetraenoyl ethanolamide	DEA	Docosatetraenoic acid (22:4n6)	NAE	0.69±0.19	0.58±0.09	0.20–1.28	0.45±0.057	0.49±0.032	0.33–0.66
Oleoyl ethanolamide	OEA	Oleic acid (18:1n9)	NAE	4.47±1.03	3.20±0.39	1.67–6.01	2.74±0.16	2.72±0.34	1.28–5.90
Stearoyl ethanolamide	SEA	Stearic acid (18:0)	NAE	9.01±0.50	9.93±0.93	5.91–18.1	9.85±1.1	10.9±0.95	7.66–19.6

**Fig 2:**
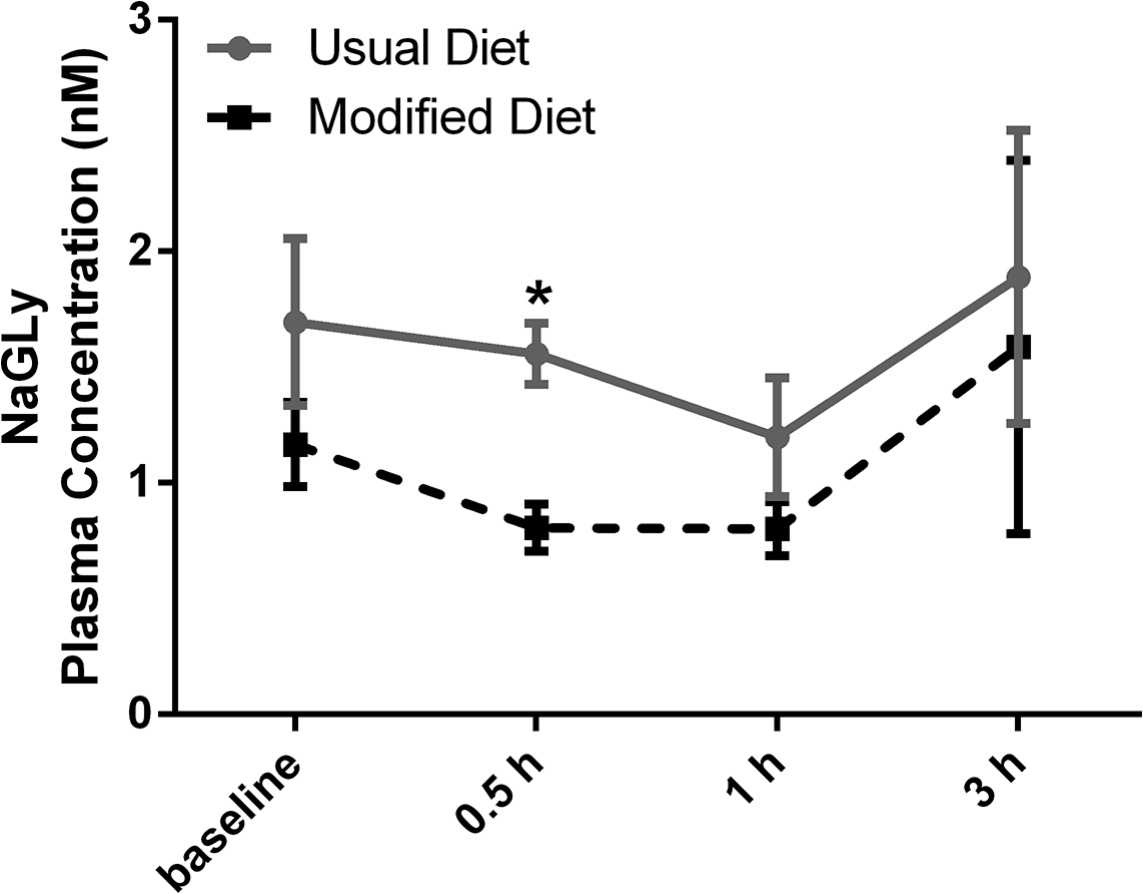
Comparison of the postprandial response in NAGly (eCB) plasma levels in one subject on usual (vegan) and modified (vegetarian) diet, respectively. *p < 0.05 (adjusted for multiple comparisons).

**Fig 3:**
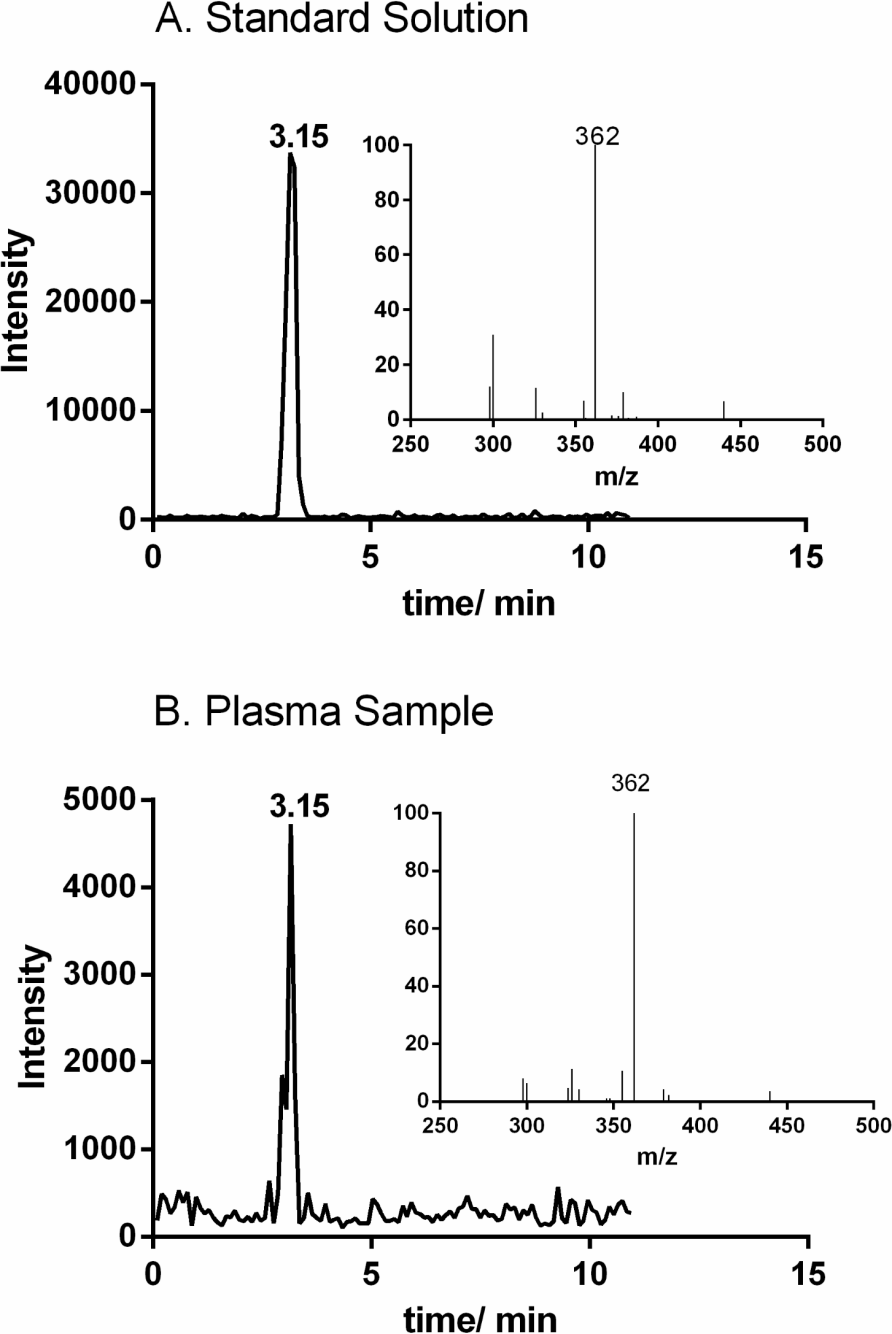
MRM chromatograms of (A) NAGLy in a standard solution (136 pg on column) and (B) a representative plasma sample together with corresponding MS spectra (inserts).

Student’s *t*-test was applied to investigate diet-dependent differences at baseline (during fasting) and in the postprandial state. There were no significant difference at baseline, and NAGly was the only compound that showed a significant diet-dependent difference in the postprandial state (at 0.5 hours after the meal, **Fig 2**). To our knowledge, this is the first report of NAGly in human plasma. MRM chromatograms and mass spectra of a plasma sample and a standard solution to verify its identity are shown in **Fig 3**.

**Fig 4:**
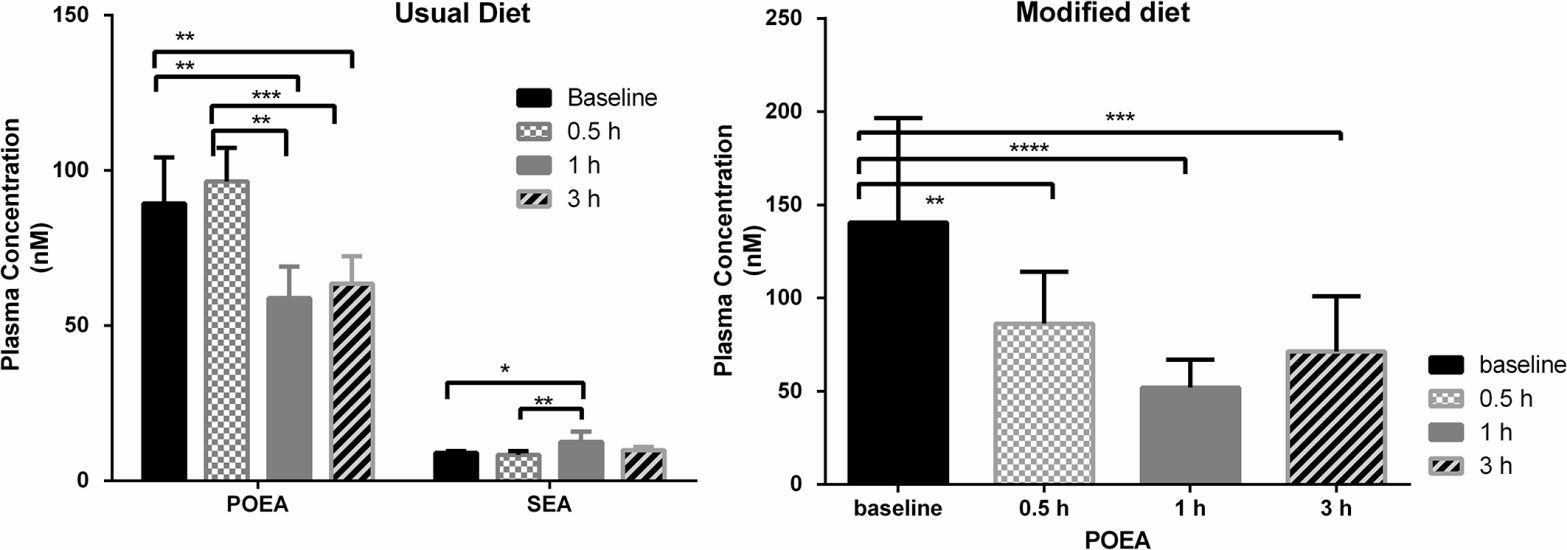
Baseline and postprandial response levels of endocannabinoid significantly different for a subject on usual diet (A), and on modified diet (B). Values represent the mean ± SEM (n = 3 for each diet and time point. ****p < 0.0001, ***p = 0.0002, **p = 0.005 (adjusted for multiple comparisons).

One-way ANOVA revealed alterations in the postprandial POEA and SEA levels when the subject was on a vegan diet (**Fig 4A**), with decreased levels of POEA at 1 and 3 hours after the meal, and increased levels of SEA at 1 hour after the meal. Only POEA postprandial levels were altered when the subject was on the modified diet (**Fig 4B**), with decreased levels at 0.5, 1 and 3 hours after the meal.

### Oxylipin quantification

**Fig 5:**
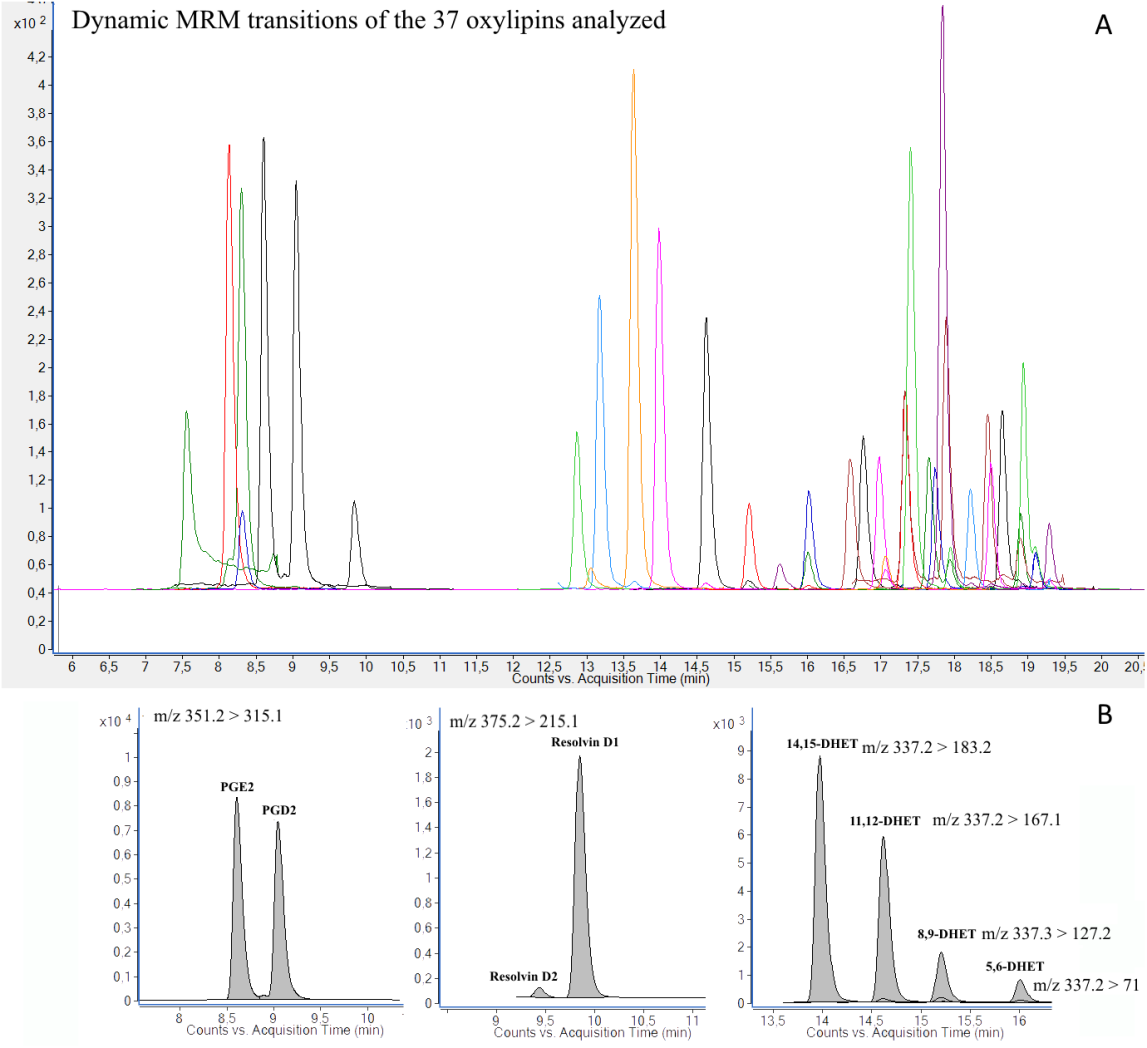
MRM chromatograms of each analyte analyzed in a standard solution mixture (A) and separation of critical pairs of isomers (B).

Dynamic MRM with defined retention time windows instead of time segments was performed allowing the instrument to monitor transitions only during the stated time window and therefore reducing the number of concurrent transitions. An extracted MRM chromatogram of all oxylipins included in the method is shown in **Fig 5**. No cross-talk between channels that were used for monitoring quantification standards, internal standards and the recovery standard was observed.

### Separation optimization

Separation of all 37 oxylipins was achieved, including critical pairs of isomers such as PGE_2_ and PGD_2_, and Resolvin D1 and D2 with the same MRM transitions (**Fig 5**). The specific detection of compounds with overlapping retention times, but different transitions is illustrated in **S2 Fig**. Eluent B acidified with 0.1% acetic acid improved signals for the prostaglandins PGD_2_, PGE_2_, PGF_2α_, and TXB_2_, but impaired the signals of the other compounds. Therefore, eluent B was not acidified in the final protocol. The optimal UPLC conditions, column temperature, gradient composition and pH were selected based on the resolution and intensity of each peak. The retention time accuracy for each compound was 99.2%±0.3% (n = 10).

#### MS/MS optimization

The most intense transition for each analyte was used for quantification purposes, marked in bold in the list of MRM transitions, collision energies and cell voltages (**Table 2**). The second transition was used as a qualifier fragment. Internal standards were assigned to each native standard according to their structural similarities and hence retention time (**S2 Table)**, with the exception of 14(15)-EET, 11(12)-EET, 5-oxo-ETE, 8(9)-EET, and 5(6)-EET that were eluting close to 12(13)-EpOME-d_4_ but were assigned to 9(S)-HODE-d_4_, because of superior linearity of the latter.

**Table 2:** Mass spectrometry parameters for multiple reaction monitoring transitions [Retention Time (RT), Cell Accelerator Voltage (CA), Collision Energy (CE)], linearity, Limit of Quantification (LOQ), and Limit of Detection (LOD) for the analyzed compounds (Fragmentor Voltage: 380 V for all compounds).

Compound	Abbreviation	rt [min]	Precursor ion	Transition	CA [V]	CE [V]	Slope	R^2^	LOD[pg on column]	LOQ[pg on column]	Linear range [pg/μL]
Thromboxane B_2_-d_4_	TXB_2_-d_4_	7.54	373.25	**373.25 < 173.00**	7	9	0.0021	0.9993			
				373.25 < 199.00	7	9					
Thromboxane B_2_	TXB_2_	7.57	369.23	**369.23 < 169.10**	7	13	0.051	0.9994	0.01	0.05	0.0053–215.31
				369.23 < 195.00	7	9					
9,12,13-trihydroxy-octadecenoic acid	9,12,13-TriHOME	8.14	329.23	**329.23 < 211.10**	5	21	0.0549	0.9976	0.0005	0.01	0.0011–215.31
				329.23 < 229.10	5	17					
9,10,13-trihydroxy-octadecenoic acid	9,10,13-TriHOME	8.31	329.23	**329.23 < 171.00**	7	21	0.0478	0.9954	0.01	0.05	0.0053–215.31
				329.23 < 139.10	7	21					
Prostaglandin F_2α_	PGF_2α_	8.33	353.23	**353.23 < 193.30**	7	21	0.009	0.9965	0.01	0.05	0.0053–215.31
				353.23 < 211.00	7	21					
Prostaglandin E_2_-d_4_	PGE_2_-d_4_	8.60	355.24	**355.24 < 319.20**	7	5	0.0031	0.9998			
				355.24 < 275.20	7	13					
Prostaglandin E_2_	PGE_2_	8.64	351.21	**351.21 < 315.10**	7	5	0.0336	0.9985		0.05	0.0053–215.31
				351.21 < 271.20	7	13					
Prostaglandin D_2_	PGD_2_	9.08	351.21	**351.21 < 315.20**	6	9	0.033	0.9987	0.26	0.53	0.053–215.31
				351.21 < 271.10	6	13					
7S,16R,17S-trihydroxy-4Z,8E,10Z,12E,14E,19Z-docosahexaenoic acid	Resolvin D2	9.45	375.21	**375.21 < 215.10**	4	13	0.0003	0.997	0.53	4.21	0.42–215.31
				375.21 < 216.10	4	13					
7S,8R,17S-trihydroxy-4Z,9E,11E,13Z,15E,19Z-docosahexaenoic acid	Resolvin D1	9.86	375.21	**375.21 < 215.10**	4	17	0.0083	0.9988	0.05	0.26	0.026–215.31
				375.21 < 217.1	4	13					
Leukotriene B_4_	LTB_4_	12.91	335.22	**335.22 < 195.10**	5	13	0.0141	0.9992	0.05	0.26	0.026–215.31
				335.22 < 317.20	5	13					
12,13-dihydroxy-octadecenoic acid-d4	12(13)-DiHOME-d4	13.15	317.26	**317.26 < 185.10**	7	21	0.0008	0.9999			
12,13-dihydroxy-octadecenoic acid	12(13)-DiHOME	13.22	313.24	**313.24 < 183.20**	7	17	0.048	0.9958	0.01	0.05	0.0053–215.31
				313.24 < 99.00	7	25					
9,10-dihydroxy-octadecenoic acid	9(10)-DiHOME	13.68	313.20	**313.20 < 201.00**	7	17	0.0852	0.9985		0.0005	0.000053–215.31
				313.20 < 59.10	7	17					
14,15-dihydroxy-eicosatrienoic acid	14,15-DHET	14.03	337.24	**337.24 < 207.00**	7	13	0.0714	0.9961	0.01	0.05	0.0053–215.31
				337.24 < 129.20	7	17					
11,12-dihydroxy-eicosatrienoic acid	11,12-DHET	14.67	337.24	**337.24 < 167.10**	7	17	0.0496	0.9974	0.05	0.26	0.026–215.31
8,9-dihydroxy-eicosatrienoic acid	8,9-DHET	15.26	337.24	**337.24 < 127.20**	4	21	0.0152	0.9976	0.05	0.26	0.026–215.31
5,6-dihydroxy-eicosatrienoic acid	5,6-DHET	15.69	319.23	3**37.24 < 71.00**	7	13	0.007	0.9974	0.05	0.26	0.026–215.31
				337.24 < 145.10	7	13					
12-hydroxy-eicosapentaenoic acid	12-HEPE	16.07	317.21	**317.21 < 179.1**	7	25	0.0177	0.9978	0.01	0.05	0.0053–215.31
				337.24 < 299.10	7	29					
20-hydroxy-eicosatetraenoic acid	20-HETE	16.09	319.23	**319.23 < 289.2**	7	9	0.0054	0.9973	0.53	1.05	0.11–215.31
				319.23 < 180.10	7	9					
13-hydroxy-octadecadienoic acid	13-HODE	16.66	295.23	**295.23 < 195.10**	6	17	0.3034	0.9961	0.05	0.26	0.026–215.31
				295.23 < 277.10	6	17					
9-hydroxy-octadecadienoic acid-d4	9-HODE-d_4_	16.76	299.25	**299.25 < 172.00**	6	29	0.0001	0.9996			
9-hydroxy-octadecadienoic acid	9-HODE	16.83	295.23	**295.23 < 171.20**	6	13	0.3385	0.9986		0.0005	0.000053–215.31
				295.23 < 277.20	6	13					
15-hydroxy-eicosatetraenoic acid	15-HETE	17.05	319.23	**319.23 < 219.00**	5	9	0.2892	0.9966	0.01	0.05	0.0053–215.31
				319.23 < 301.20	5	5					
17(R)-hydroxy-docosahexaenoic acid	17(R)-HDoHE	17.13	343.23	**343.23 < 281.20**	7	9	0.0932	0.9944	0.53	1.05	0.11–215.31
				343.23 < 201.30	7	9					
13-oxo-octadecadienoic acid	13-oxo-ODE	17.14	293.21	**293.21 < 113.10**	7	5	0.0412	0.9981	1.05	4.21	0.42–215.31
				293.21 < 165.20	6	17					
15-oxo-eicosatetraenoic acid	15-oxo-ETE	17.40	317.21	**317.21 < 113.20**	6	13	0.4556	0.9973	0.05	0.26	0.026–215.31
				317.21 < 273.10	6	9					
11-hydroxy-eicosatetraenoic acid	11-HETE	17.47	319.23	**319.23 < 167.20**	6	9	0.07352	0.9978		0.01	0.0011–215.31
12-hydroxy-eicosatetraenoic acid	12-HETE	17.73	319.23	**319.23 < 179.10**	6	9	0.327	0.9987	0.05	0.26	0.026–215.31
8-hydroxy-eicosatetraenoic acid	8-HETE	17.80	319.23	**319.23 < 155.00**	6	9	0.2639	0.9993	0.53	1.05	0.11–215.31
				319.23 < 301.20	6	9					
15-hydroxy-eicosatrienoic acid	15(S)-HETrE	17.90	321.24	**321.24 < 303.30**	6	9	1.2883	0.9988	0.0005	0.01	0.0011–215.31
				321.24 < 221.10	6	13					
12-oxo-eicosatetraenoic acid	12-oxo-ETE	17.95	317.21	**317.21 < 273.30**	4	9	0.6082	0.9958	0.01	0.05	0.0053–215.31
				317.21 < 153.20	4	13					
9-hydroxy-eicosatetraenoic acid	9-HETE	18.01	319.23	**319.23 < 167.20**	4	9	0.076	0.9996	2.1	4.21	0.42–215.31
				319.23 < 123.10	4	17					
5-hydroxy-eicosatetraenoic acid	5-HETE	18.28	319.23	**319.23 < 115.10**	4	13	0.2269	0.997	0.05	0.26	0.026–215.31
				319.23 < 301.10	4	5					
12(13)epoxy-octadecenoic acid-d_4_	12(13)-EpOME-d_4_	18.44	299.25	**299.25 < 281.00**	4	4	0.0002	0.9997			
12(13)epoxy-octadecenoic acid	12(13)-EpOME	18.52	295.23	**295.23 < 195.10**	5	13	0.127	0.9938	0.26	0.53	0.053–215.31
				295.23 < 277.20	5	13					
14(15)-epoxy-eicosatrienoic acid	14(15)-EET	18.54	319.22	**319.22 < 219.00**	5	5	0.2564	0.9983	0.01	0.05	0.0053–215.31
				319.22 < 301.00	5	5					
9(10)epoxy-octadecenoic acid	9(10)-EpOME	18.72	295.23	**295.23 < 171.20**	4	13	0.1252	0.9969	0.26	0.53	0.053–215.31
5-oxo-eicosatetraenoic acid	5-oxo-ETE	19.00	317.23	**317.23 < 203.20**	4	13	0.1555	0.9968	0.05	0.26	0.026–215.31
				317.23 < 59.10	4	21					
11(12)-epoxy-eicosatrienoic acid	11(12)-EET	18.96	319.23	**319.23 < 167.10**	4	9	0.4723	0.9986	0.01	0.05	0.0053–215.31
				319.23 < 301.2	4	5					
8(9)-epoxy-eicosatrienoic acid	8(9)-EET	19.17	319.23	**319.23 < 69.20**	4	13	0.0773	0.9995	ND	0.26	0.026–215.31
				319.23 < 123.00	4	5					
5(6)-epoxy-eicosatrienoic acid	5(6)-EET	19.34	319.23	**319.23 < 191.10**	4	5	0.1049	0.9996	0.26	0.53	0.053–215.31

Strassburg et al. [47] developed a method to analyze 104 oxylipins in a single analytical run using similar equipment to ours (without the iFunnel technology). Their 104-oxylipin method includes oxylipins that are also included in our method, with the exception of Resolvin D1 and D2. They used the same transitions as the ones reported here, except for PGE_2_, 5,6-DHET, 12-HEPE, 13-HODE, 9-HODE, 15-HETE, 5-HETE, and 12(13)-EpOME.

#### Linearity, LOD and LOQ

The linearity of the method was determined with calibration curves over a concentration range of 0.05–2200 pg on column. Regression analysis without weighing factors produced R^2^ values between 0.9938 and 0.9996 (**Table 2**). The LOD ranged between 0.0005 and 2.1 pg on column, while LOQ ranged between 0.0005 and 4.2 pg on column (**Table 2**). Yang et al. [46] established LOQ values between 0.03 and 15.96 pg on column. Their LOQs for 15-oxo-ETE, 9-HETE, 11,12-DHET, and LTB_4_ were lower compared to ours. For all other compounds their LOQ values were equal or higher than ours. A comparison of all individual LOQs can be found in **S6 Table**.

Wang et al. [48] recently reported a comprehensive method for analysis of eicosanoids and other bioactive lipids in human plasma (184 compounds). However, the 35 compounds in common with our method presented higher LOQ values (**S6 Table**). Furthermore, a combined method to quantify prostanoids and *N*-acylethanolamines in human plasma included a sub-set of the compounds in our method, but also with higher LOQ values [49].

Despite the advantage illustrated above of a large panel of compounds analyzed with a short run time [26, 48, 49], a compromised sensitivity is unavoidable with current MS equipments. Therefore, we decided to select a sub-set of compounds including those previously reported in human plasma and others of potential interest to postprandial inflammation.

#### Accuracy and precision

Four QC samples at different concentrations were used to establish accuracy and precision of the method (**S7 Table**). In the current study, the sample on-column amounts were in general close to QC3 and QC4, and occasionally at the level of QC2. Precision ranged from 0.1 to 17%, and were in general 5% or below (for 3/4 of the samples). Only 1/20 of the QC samples displayed CV values of 10% or higher. Hence, conditions for a precise method were achieved for all oxylipins tested according to the FDA guidelines of CV below 20%. Furthermore, the majority of QC samples (4/5) were in the range of 85 to 115%, thereby fulfilling the FDA guidelines of accuracy (80–120%). However, the accuracy ranged from 48 to 175% at the lowest QC level for 5 compounds (9,10,13-TriHOME, PGF2α, 12(13)-EPOME, 5(6)-EET and 12-oxo-ETE).

#### Recovery

**Fig 6:**
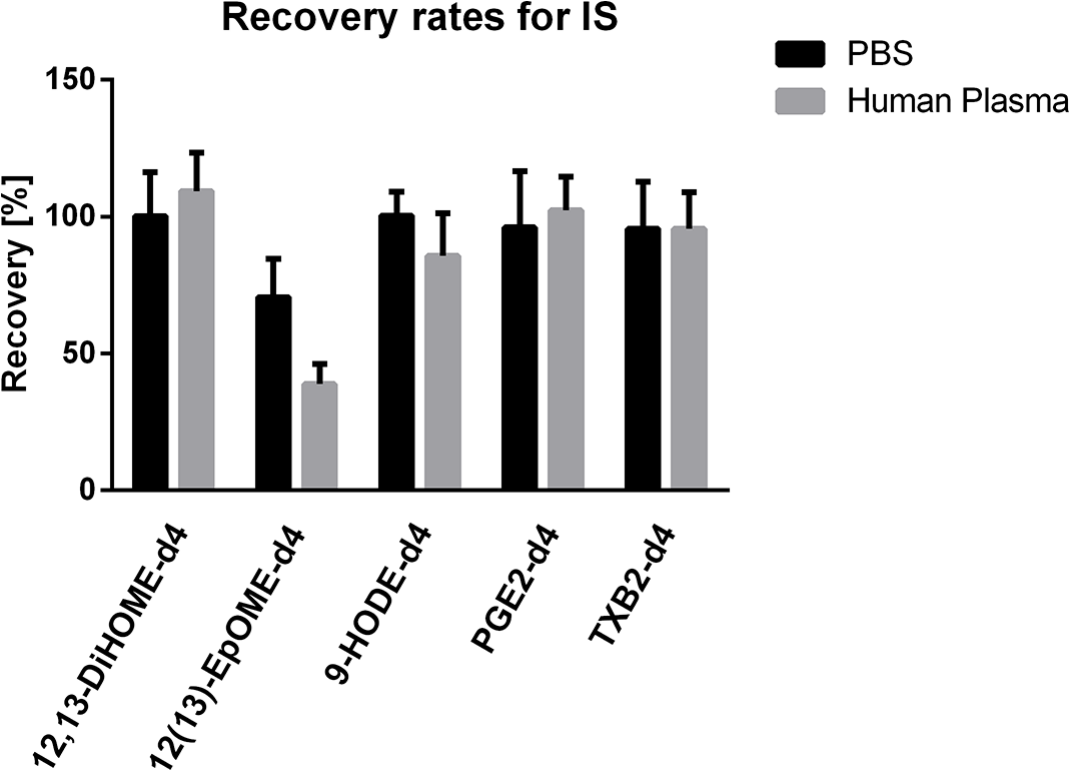
Average recovery rates for deuterated internal standards (IS) in PBS (n = 5) and human plasma (n = 24) Expressed as mean±SEM.

The average recovery rates ranged from acceptable 71 to 109% in PBS, human, fish and pig plasma, as well as CSF (**Fig 6**), except for the recovery of 12(13)-EpOME-d_4_ in plasma, which was approximately 40%. The recovery for internal standards was considered representative of the recovery for the native compounds, thus good recovery rates for internal standards ensure well-functioning extraction and analysis of the endogenous oxylipins. It has previously been shown that the polarity of each compound influences the recovery from different matrices according to their lipophilicity and/or presence of protein [51]. Our results confirm PBS to mimic human plasma with similar recovery rates for the internal standards.

#### Stability

Stability tests of human plasma analyzed after 7, 31 and 300 days in -80°C showed that the oxylipins were stable over the whole period (up to 300 days) with the exception of 15-oxo-ETE (**S8 Table**). Two cycles of freeze-thaw (at -20°C for one week) resulted in a higher degree of degradation when compared to the longest storage time at -80°C. The stability values in human plasma for the endocannabinoids and related compounds were in line with those reported in bovine milk [34].

#### Analysis of changes in metabolites

In the 24 human plasma samples analyzed, 35 oxylipins derived from 4 different PUFAs and biosynthesized *via* three different pathways (COX, LOX, and CYP) could be detected (**Table 3**). These results are in accordance to other studies, which also quantified a variety of oxylipins in human plasma [27, 47]. Twenty-six oxylipins were detected in all samples and at all-time points independent of background diet (both usual and modified diets), 20-HETE was detected in all samples on usual diet and 7/12 of modified diet samples, 8(9)-EET, 11(12)-EET, and 5-oxo-ETE were detected in all except one or two samples, while 5 oxylipins (LTB4, 5,6-DHET, 17(R)-HDoHE, 9-HETE and 14(15)-EET were detected irregularly (**S9 Table**). Two compounds, Resolvin D1 and 2, were not detected at all. Measurable concentrations of Resolvin D1 and 2 were, however, previously found in human plasma following ω3 fatty acid supplementation [52]. Resolvins are known to act as lipid mediators in the resolution of inflammation [53] and might therefore not be present, or only at very low concentrations, in plasma of a healthy subject.

**Table 3:** Average levels (nM)±SEM of compounds in the oxylipin metabolome of human plasma at fasting (baseline) and in the fasting and postprandial state (all samples) in a subject on her usual or modified diet.

				Usual Diet			Modified Diet		
Oxylipin	Fatty acid precursor	Chemical class	Pathway	Baseline (n = 3)	All samples (n = 12)	Range	Baseline (n = 3)	All samples (n = 12)	Range
TXB_2_	AA	Triol	COX	1.16±0.052	0.91±0.1	0.26–1.3	0.39±0.073	0.86±0.14	0.22–1.69
9,12,13-TriHOME	LA	Triol	5-LOX	0.72±0.028	0.82±0.064	0.51–1.3	1.92±1.2	1.34±0.34	0.47–4.41
9,10,13-TriHOME	LA	Triol	5-LOX	0.96±0.008	1.08±0.078	0.75–1.6	2.65±1.7	1.96±0.45	0.72–6.03
PGF_2α_	AA	Triol	COX	0.30±0.045	0.36±0.023	0.22–0.52	0.44±0.015	0.38±0.018	0.30–0.47
PGE_2_	AA	Diol/Ketone	COX	0.25±0.030	0.21±0.012	0.15–0.30	0.18±0.016	0.21±0.01	0.14–0.26
PGD_2_	AA	Diol/Ketone	COX	0.05±0.012	0.04±0.005	0.02–0.08	0.05±0.005	0.05±0.003	0.04–0.07
12(13)-DiHOME	LA	Diol	CYP	9.61±1.2	6.77±0.70	3.35–11.9	5.30±1.7	3.70±0.61	1.22–8.68
9(10)-DiHOME	LA	Diol	CYP	6.02±0.41	4.26±0.43	2.05–6.78	3.59±1.4	2.41±0.48	0.62–6.29
14,15-DHET	AA	Diol	CYP	0.57±0.11	0.42±0.038	0.29–0.78	0.30±00.055	0.28±0.021	0.18–0.37
11,12-DHET	AA	Diol	CYP	0.31±0.071	0.21±0.024	0.14–0.45	0.13±0.010	0.12±0.008	0.07–0.19
8,9-DHET	AA	Diol	CYP	0.13±0.030	0.10±0.009	0.07–0.19	0.09±0.008	0.08±0.006	0.04–0.11
5,6-DHET	AA	Diol	CYP	*ND*	0.01±0.008	*ND*	*ND*	0.01±0.012	0.00–0.14
12(S)-HEPE	EPA	Alcohol	15-LOX	0.06±0.05	0.05±0.006	0.02–0.10	0.07±0.033	0.09±0.017	0.03–0.23
20-HETE	AA	Alcohol	CYP	0.23±0.077	0.16±0.024	0.05–0.37	0.06±0.057	0.06±0.021	0.00–0.22
13-HODE	LA	Alcohol	5-LOX	17.35±1.2	12.13±1.5	4.34–19.67	30.8±22	11.85±5.8	3.46–75.81
9-HODE	LA	Alcohol	5-LOX	12.64±1.1	7.82±1.07	2.92–13.95	19.8±15	7.24±4.0	1.92–51.08
15-HETE	AA	Alcohol	15-LOX	0.73±0.13	0.52±0.063	0.19–0.97	0.87±0.57	0.52±0.14	0.25–2.02
17-HDoHE	DHA	Alcohol	15-LOX	*ND*	0.03±0.035	0.00–0.42	1.11±1.1	0.28±0.28	0.00–3.32
13-oxo-ODE	LA	Ketone	5-LOX	6.12±0.67	3.73±0.60	1.18–7.26	10.69±9.4	3.39±2.4	0.27–29.46
11-HETE	AA	Alcohol	15-LOX	0.25±0.034	0.17±0.023	0.06–0.32	0.23±0.13	0.16±0.032	0.08–0.49
15-oxo-ETE	AA	Ketone	15-LOX	0.17±0.027	0.12±0.014	0.05–0.21	0.38±0.32	0.16±0.079	0.04–1.02
12-HETE	AA	Alcohol	15-LOX	0.79±0.12	0.58±0.10	0.16–1.30	0.41±0.17	0.71±0.17	0.18–2.02
8-HETE	AA	Alcohol	15-LOX	0.20±0.042	0.12±0.018	0.07–0.28	0.17±0.10	0.10±0.026	0.04–0.36
15(S)-HETrE	AA	Alcohol	15-LOX	0.13±0.031	0.11±0.015	0.06–0.23	0.20±0.16	0.11±0.038	0.03–0.52
12-oxo-ETE	AA	Ketone	15-LOX	0.82±0.053	0.73±0.046	0.44–0.93	0.75±0.13	0.69±0.033	0.55–0.99
9-HETE	AA	Alcohol	15-LOX	0.07±0.005	0.03±0.012	0.00–0.10	0.07±0.072	0.06±0.018	0.00–0.22
5-HETE	AA	Alcohol	5-LOX	0.30±0.069	0.19±0.028	0.08–0.43	0.46±0.33	0.20±0.085	0.06–1.13
12(13)-EpOME	LA	Epoxide	CYP	1.74±0.19	1.28±0.17	0.61–2.31	1.80±1.1	0.75±0.30	0.10–3.92
14(15)-EET	AA	Epoxide	CYP	0.05±0.017	0.03±0.007	0.00–0.08	0.07±0.046	0.03±0.013	0.00–0.16
9(10)-EpOME	LA	Epoxide	CYP	3.67±0.49	2.54±0.28	1.04–4.62	4.30±2.9	1.74±0.77	0.46–10.19
11(12)-EET	AA	Epoxide	CYP	0.13±0.027	0.09±0.013	0.00–0.18	0.15±0.035	0.11±0.012	0.06–0.20
5-oxo-ETE	AA	Ketone	5-LOX	0.06±0.010	0.06±0.012	0.00–0.16	0.19±0.24	0.09±0.040	0.00–0.52
8(9)-EET	AA	Epoxide	CYP	0.11±0.032	0.07±0.012	0.02–0.17	0.13±0.095	0.07±0.024	0.00–0.32
5(6)-EET	AA	Epoxide	CYP	0.25±0.056	0.14±0.024	0.06–0.30	0.28±0.14	0.13±0.040	0.02–0.55

AA – Arachidonic acid (20:4n6)); LA – Linoleic acid (18:2n6); DHA – Docosahexaenoic acid (22:4n6); EPA – Eicosapentaenoic acid (20:5n3); ND – Not detected.

Oxylipins above LOQ and with S/N > 10 were included in the statistical calculations of the postprandial response. For oxylipins with S/N > 10 (but levels below LOQ), the concentration was calculated using an extrapolated calibration curve. For oxylipins with S/N between 3 and 10 (and levels below LOQ), the value of LOD was used. In total, the variability of 35 oxylipins was investigated.

Oxylipin levels ranged over three orders of magnitude from 0.01 to 76 nM, where baseline levels on usual diet ranged from 0.03 to 19 nM and modified diet baseline levels ranged from 0.03 to 76 nM (**Table 3**).

**Fig 7:**
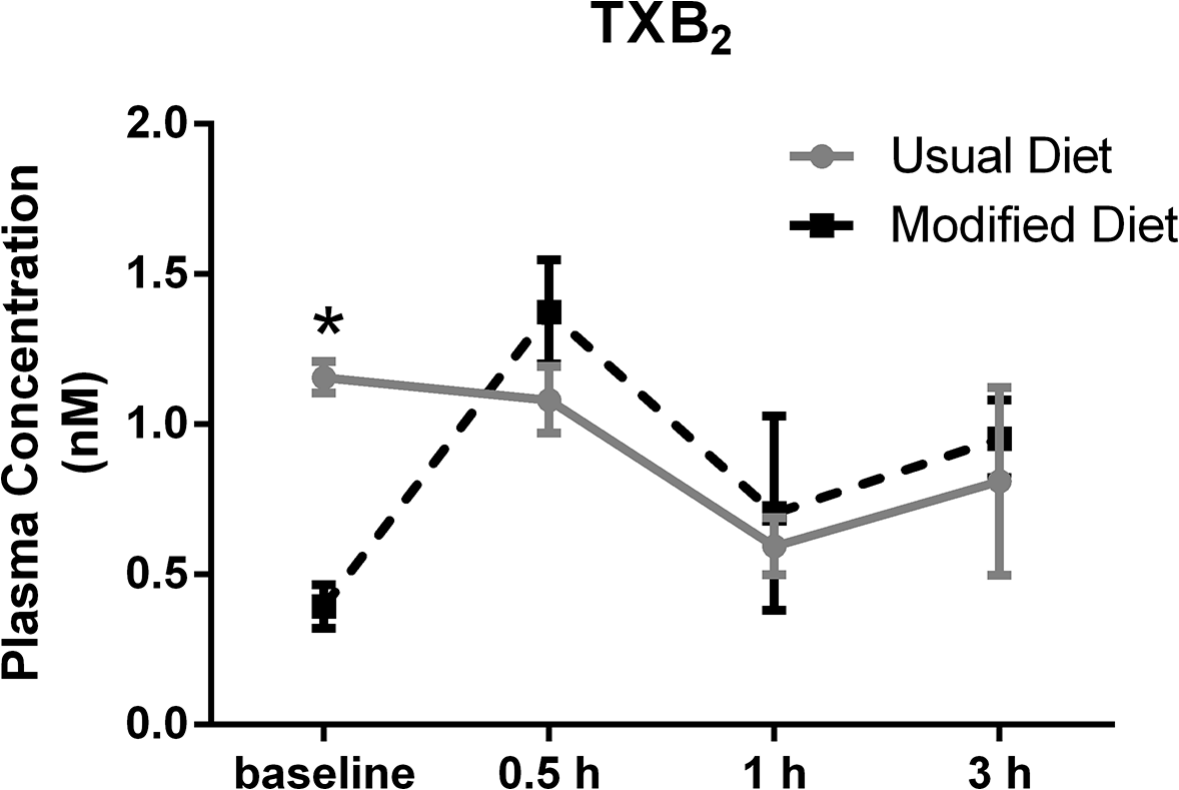
Significant different baseline levels of TXB_2_ (p = 0.001) in plasma collected at usual (n = 3) and modified (n = 3) background diets, respectively. Values represent the mean ± SEM.

Of all oxylipins tested, only one compound (TXB_2_) displayed significantly different baseline levels between the different background diets. There were decreased TXB_2_ levels when the subject was on the modified diet compared to the usual background diet (**Fig 7**). TXB_2_ is an inactive COX product of arachidonic acid (AA, 20:4n6). Its precursor, TXA_2_, is the major COX product in blood platelets and acts as a pro-aggregator and vasoconstrictor [54]. According to Sinclair et al [55], diets rich in AA show no effect on levels of TXA_2_, suggesting that differences in TXB_2_ levels are not caused by dietary AA changes. In contrast, a connection between AA concentration in plasma and TXB_2_ production in phenylketonuric subjects and lower concentrations of TXB_2_ in serum was found [56].

**Fig 8:**
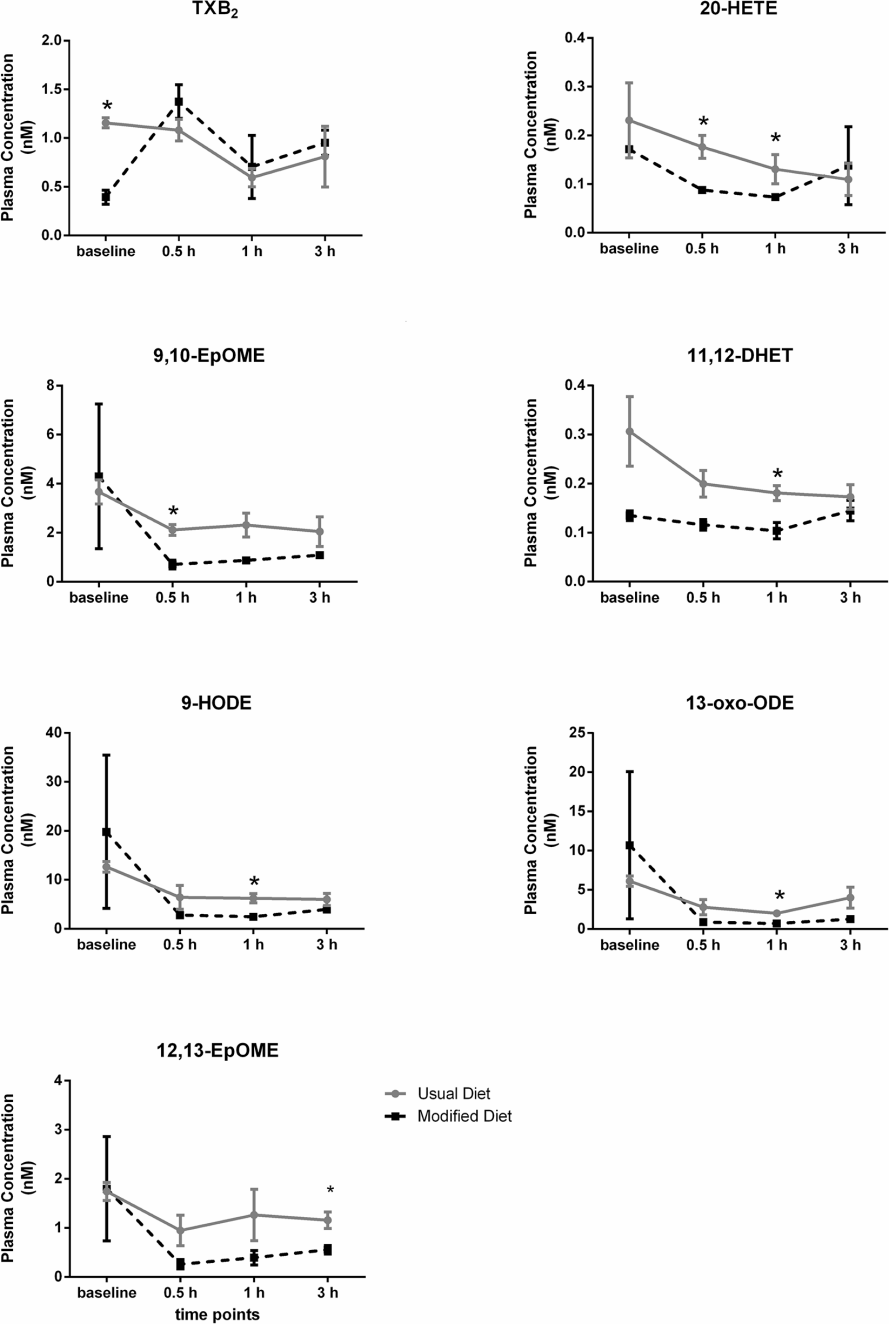
Comparison of the postprandial oxylipin plasma levels in one subject on a usual and modified diet, respectively. * indicates p < 0.05 (adjusted for multiple comparisons).

Beside the TXB_2_ decrease in baseline oxylipin levels due to a modified background diet, an additional six oxylipins showed significantly lower levels at one or more time points during the postprandial state when the subject was on the modified diet compared to the usual background diet (**Fig 8)**. Notably, TXB_2_ showed no significant differences between background diets in the postprandial state, only at baseline. During the postprandial response, two linoleic acid (LA, 18:2n6) products from the LOX pathway (9-HODE and 13-oxo-ODE), showed significant differences at one hour after the meal between the usual and modified background diets. Furthermore, significantly different levels were detected in four CYP- derived oxylipins; both from the LA precursor (9(10)-EpOME and 12(13)-EpOME), and the AA precursor (20-HETE and 11,12-DHET).

**Fig 9:**
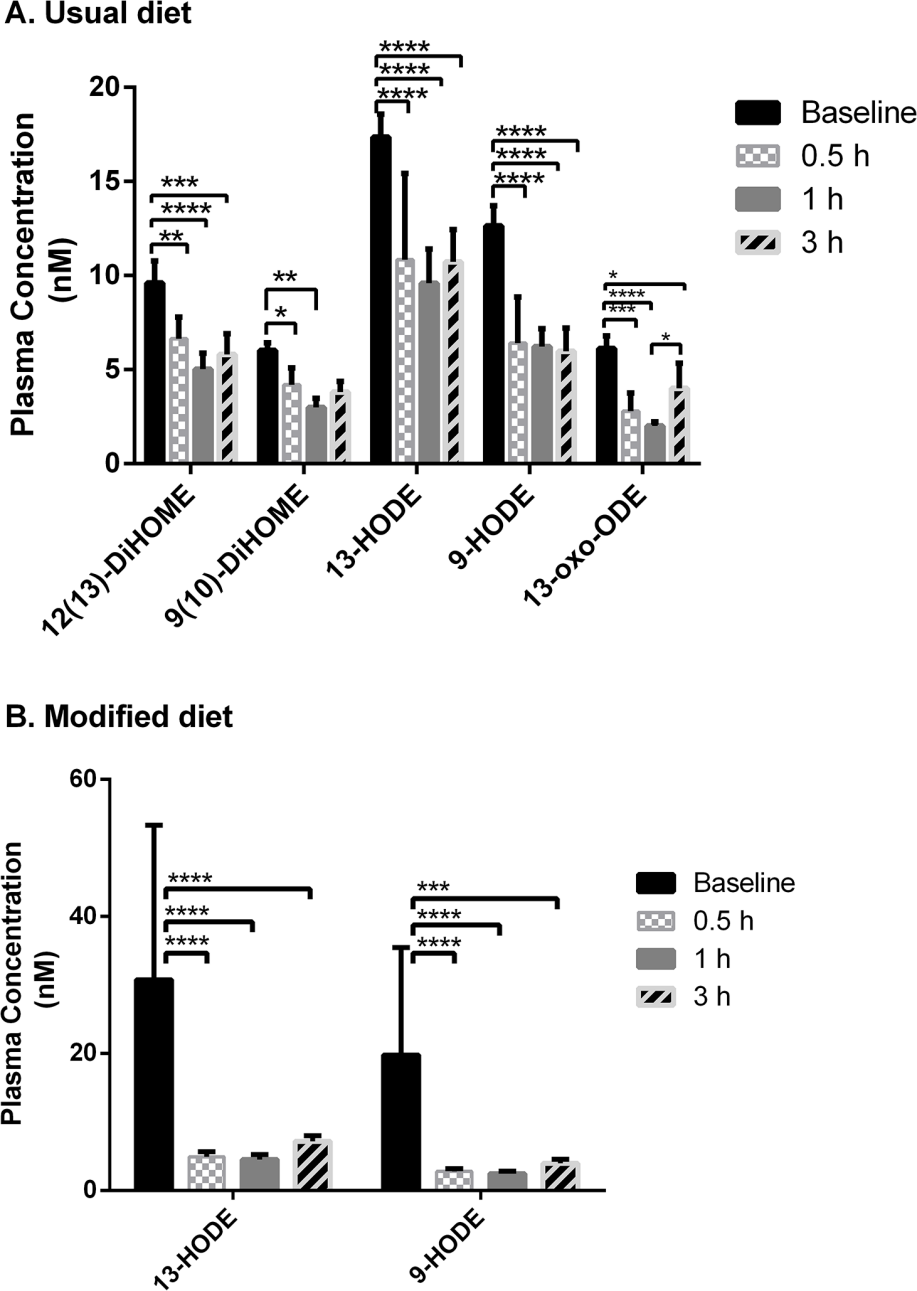
Baseline and postprandial response levels significantly different of oxylipins for a subject on usualdiet (A), and on modified diet (B). Values represent the mean ± SEM (n = 3 for each diet and time point). Brackets indicates significantly different time points: ****p < 0.0001, ***p = 0.0002 (adjusted for multiple comparisons).

One-way ANOVA revealed significant differences across the time points in the postprandial oxylipin response (**Fig 9)**. Alterations in the postprandial oxylipin levels were observed only for compounds derived from LA. For the diols, biosynthesized *via* the CYP pathway, a significant decrease was visible in vegan samples at 1 hour (9(10)-DiHOME) and at 0.5, 1, and 3 hours (12(13)-DiHOME) after the meal compared to the baseline level (at fasting). Furthermore, the ketone 13-oxo-ODE from the LOX pathway also displayed a significant decrease at 0.5 and 1 hour after the meal compared to the baseline level in usual diet samples. Other LOX products, 9-HODE and 13-HODE (alcohols), were found to also decrease in comparison to baseline for both usual and modified diet samples at 0.5, 1, and 3 hours after the meal. No significant changes, however, were observed for COX-derived metabolites (e.g. prostaglandins).

### Profiling of the oxylipin and endocannabinoid metabolome

**Fig 10:**
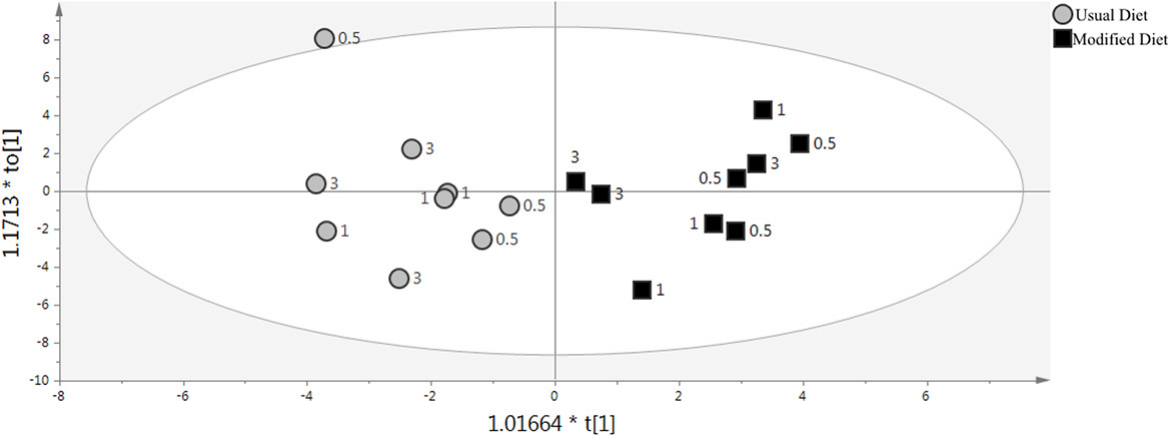
Oxylipin and endocannabinoid profiles in the postprandial state recapitulated as orthogonal scores (t[1] and to[1]) calculated by OPLS-DA. Time after challenge meal (in hours) is found next to each sample. The subject displayed different oxylipin and endocannabinoid metabolomes on usual (grey circles) vs modified (black squares) background diet. Model assessment parameters were: 1 predictive and 1 orthogonal component, p-value calculated by CV-ANOVA: 0.033, total systematic variation among the metabolites captured by the model (R2X): 0.463, total systematic variation between the diets captured by the model (R2Y): 0.815, predictive ability of the model (Q2): 0.53.

Coefficient of variation (CV) values for the oxylipins, NAEs, MAGs and related compounds in human plasma (**S3 and S4 Figs** were considerably larger than those found for quality control samples of standards spiked to PBS (**S10** and **S11 Tables**). Hence, the variability of the plasma metabolites was attributed to biological variation rather than analytical. For the purpose of investigating if the biological variation was due to noise, or if there was a true signal from the background diet, multivariate data analysis was performed combining the information from all oxylipins found in all samples (n = 26), and NAEs, MAGs and related compounds found in all but one or two samples (n = 11). We chose a conservative approach and included only the metabolites in common between all samples (but one or two) to ensure that the same metabolic domain was captured for all samples. The relationship between systematic variability and background diet (usual and modified) was investigated using multivariate regression analysis in terms of orthogonal projections to latent structures discriminant analysis (OPLS-DA) [57]. When data from only baseline samples were included (n = 6), the result was no significant OPLS-DA model assessed by ANOVA based on the cross-validated score vectors (CV-ANOVA: 0.764) [58]. The reason might be the low number of samples, however, adding all samples to the model (n = 24), resulted also in no significant model (CV-ANOVA: 0.267). On the contrary, only using data from the samples in the postprandial state (n = 18) resulted in a significant separation between samples collected during the usual and modified background diets (**Fig 10**). This was in agreement with the univariate data analysis, with only one metabolite showing a significant difference at baseline (TXB_2_), while 7 (NAGLy, 9-HODE, 13-oxo-ODE, 9(10)-EpOME, 12(13)-EpOME, 20-HETE, and 11,12-DHET) were different between the background diets in the postprandial state. However, the majority of the metabolites showed no difference due to background diet in univariate analysis, and among the ones responsive to the challenge meal, several metabolites were in common between the background diets (POEA, 9-HODE, and 13-HODE), highlighting the additional value of using multivariate data analysis in nutritional studies, which also previously has been recommended and frequently applied [59]. Furthermore, from the multivariate analysis we could conclude that metabolites in the oxylipin metabolome were more specific to each background diet than metabolites in the endocannabinoid metabolome, since only oxylipins displayed variable importance above 1, the limit for variable influence on projection considered to be relevant for separation of classes (**S12 Table**). LA derived epoxides in the CYP pathway (9(10)-EpOME and 12(13)-EpOME) were characteristic of the usual diet, and could be due to the higher levels of LA in the diet during this time period. On the other hand, an eicosapentaenoic acid derived oxylipin in the LOX pathway (12(S)-HEPE) dominated when the subject was on the modifieddiet in relation to the whole oxylipin and eCB metabolome under study (**S5 Fig**). This was despite the fact that intake of eicosapentaenoic acid, or its precursor, alpha-linolenic acid, were not different between the two diets.

To summarize, our results from the multivariate analysis imply that 1) combined data on the metabolite levels is more informative than each metabolite one by one, and 2) data on the oxylipin and endocannabinoid metabolome from the postprandial state contain more information than from the fasting state alone.

## Discussion

This study did not attempt to relate changes in diet (vegan to vegetarian) to biological markers. The change in background diet rather represented a venue to investigate the performance of the UPLC-ESI-MS/MS methods to detect subtle fluctuations among the bioactive lipids, in this case introduced by the difference in diets, within a single subject tested with the challenge meal study design at multiple occasions. Thereby, the investigation served as a pilot study in which the biological variability was assessed and compared to the analytical variability of the UPLC-ESI-MS/MS methods. From our multivariate analysis it was evident that inclusion of dairy products in the diet produced a shift in bioactive lipid profiles in the postprandial state (**Fig 10**), most notably among the oxylipins (**S5 Fig** and **S8 Table**). This shift was not present to the same extent in the fasting state alone suggesting that a subtle change in metabolite levels occurred in the postprandial state (which was successfully detected with our analytical protocols). Indeed, there was one analyte in the endocannabinoid metabolome (NAGly) and 6 in the oxylipin metabolome (20-HETE, 9,(10)-EpOME, 11,12-DHET, 9-HODE, 13-oxo-ODE and 12(13)-EpOME) that were significantly different in the postprandial state, while only one (TXB_2_) was different in the fasting state, and not in the postprandial state (**Fig 2** and **Fig 8**). So, the shift seemed to be analyte-dependent, some diverged in the postprandial state, while others converged between vegan and vegetarian diets. At the same time, there were similarities in metabolite responsiveness between the usual and modified diets. Two NAEs (POEA and SEA) and 5 oxylipins (9(10)-DiHOME, 12(13)-DiHOME, 9-HODE, 13-HODE and 13-oxo-ODE) were responsive to the challenge meal when the subject was on the usual diet, of which 1 NAE (POEA) and 2 oxylipins (9-HODE and 13-HODE) also were responsive when the subject was on a vegetarian diet (**Fig 4** and **Fig 9**). Whether the discrepancy in responsiveness is due to more variable data produced by the modified diet remains to be tested. Our data suggest that this might be the case, since higher CVs, especially at fasting, were displayed for the samples collected at modified diet compared to the usual diet (**S3 and S4 Figs**). But since only one subject was included in this pilot study, no statement can be made on inter-individual variations in order to assess the observed variations between the vegan and vegetarian diet.

Furthermore, the study design does not clarify if the variability was caused by true metabolic differences between the usual and modified diet, or if daily variations in diet and lifestyle that are independent of specific vegan and vegetarian aspects had an impact. We deliberately refrained from instructions that would limit lifestyle etc, in order to investigate how well our UPLC-ESI-MS/MS methods perform for assessment of the oxylipin and endocannabinoid metabolome in a free-living individual. However, to gain better understanding of dietary effects on bioactive lipid profiles, it is necessary to carry out further comprehensive studies, including additional endpoints, on more subjects. For instance, it would be valuable to follow the postprandial state over a prolonged time course (more than 3 hours) and also follow for instance the insulin levels. We selected 3 hours since it represents the lipemic peak [60]. The experiment was stopped at 3 hours since the challenge meal only provided 15% of daily calories. But with another type of challenge meal, it would be possible to prolong the experiment.

In addition to the 3 hour time point, we also measured the bioactive lipid levels in the early postprandial state, since we hypothesized that the metabolites under study would be fast responders to the meal intake. This proved to be true, since all of the postprandial responses were detected within 1 hour after the meal intake, and all except 3 out of 10 persisted until 3 hours after the meal intake (**Fig 4** and **Fig 9**). Interestingly, all metabolites (except SEA) responded by decreasing levels, which might be a reflection of the low content of precursor fatty acids in the challenge meal (banana). Whether this is characteristic of postprandial inflammation or attributed to a normal homeostatic response remains to be tested. Our data confirms that the oxylipin and endocannabinoid metabolome are responsive to a challenge meal and that small fluctuations in their concentrations can be detected and quantified reproducibly. In addition, we found that multivariate analysis highlighted the subtle diet-dependent changes in the postprandial state. Therefore, UPLC-ESI-MS/MS analysis of these metabolites using a well-validated method can be used to investigate subtle changes expected in lipid-mediated inflammation in connection to meal intake and other clinical conditions and settings where subtle variations are important to detect.

## Supporting Information

S1 Fig Chemical structures of endocannabinoid and oxylipin analytes under study. (TIF)

S2 Fig Unique quantification of analytes with overlapping retention times but different MRM transitions. (TIF)

S3 Fig Coefficient of variation (CV) for oxylipins in plasma from a subject on usual (left panel) and modified (right panel) diet at baseline (fasting) and in the postprandial state (0.5, 1, and 3 hours after the challenge meal). (TIF)

S4 Fig Coefficient of variations (CV) for endocannabinoids and related compounds in plasma from a subject on usual (left panel) and modified (right panel) diet at baseline (fasting) and in the postprandial state (0.5, 1, and 3 hours after the challenge meal). (TIF)

S5 Fig The corresponding loading plot to the score plot in Fig 9, showing the contribution of each metabolite to the separation due to different metabolite profiles in the postprandial state of samples collected at different background diets. Usual diet-specific metabolites are found to the left (the most typical were 9(10)-EpOME and 12(13)-EpOME, highlighted in grey), and modified diet-specific metabolites are found to the right (the most typical was 12(S)-HEPE, highlighted in grey).
(TIF)

S1 Table Mean values from seven-day dietary records for the subject on usual and modified background diets (assessed by two-tailed Student’s *t*-test, if unequal variance, p-value is for *t*-test assuming unequal variance or Welch's non-parametric test). P-values below 0.05 are highlighted in bold.
(DOCX)

S2 Table Internal standards used for quantification. (DOCX)

S3 Table Calibration standard concentrations (µg/mL) for endocannabinoids (stock solution 250 µg/mL). (DOCX)

S4 Table Calibration standard concentrations (pg/mL) for oxylipins. (DOCX)

S5 Table Endocannabinoid levels (nM) in the plasma samples from the challenge meal study in the fasting state (0) and postprandial state (at 0.5, 1, 3 hours after the meal) at six different occasions (a–f). (DOCX)

S6 Table Comparison of limit of quantification (LOQ) values with Yang *et al* [44] and Wang *et al* [48]. (DOCX)

S7 Table Inter- and intraday accuracy and precision (coefficient of variation) for quality control (QC) samples at four different concentration levels. (DOCX)

S8 Table Levels (nM) and stability of 5 pooled human plasma samples (expressed as % of the 0 day value) for the metabolites (oxylipins, endocannabinoids and related compounds) under study. (DOCX)

S9 Table Oxylipin levels (nM) in the plasma samples from the challenge meal study in the fasting state (0) and postprandial state (at 0.5, 1, 3 hours after the meal) at six different occasions (a – f). (DOCX)

S10 Table Coefficient of variation (CV) values (%) for oxylipins in the fasting state and postprandial state (at 0.5, 1, 3 hours after the meal), and in quality control (QC) samples. (DOCX)

S11 Table Coefficient of variation (CV) values (%) for endocannabinoids in the fasting state and postprandial state (at 0.5, 1, 3 hours after the meal), and in quality control (QC) samples [32]. (DOCX)

S12 Table Variable influence on projection (VIP) values for each metabolite in the OPLS-DA model. (DOCX)
