# Supplementary figures and images for: Profiling the Oxylipin and Endocannabinoid Metabolome by UPLC-ESI-MS/MS in Human Plasma to Monitor Postprandial Inflammation
Source: PLoS One. 2015 Jul 17;10(7):e0132042. doi: 10.1371/journal.pone.0132042 (PMC4506044; doi:10.1371/journal.pone.0132042)

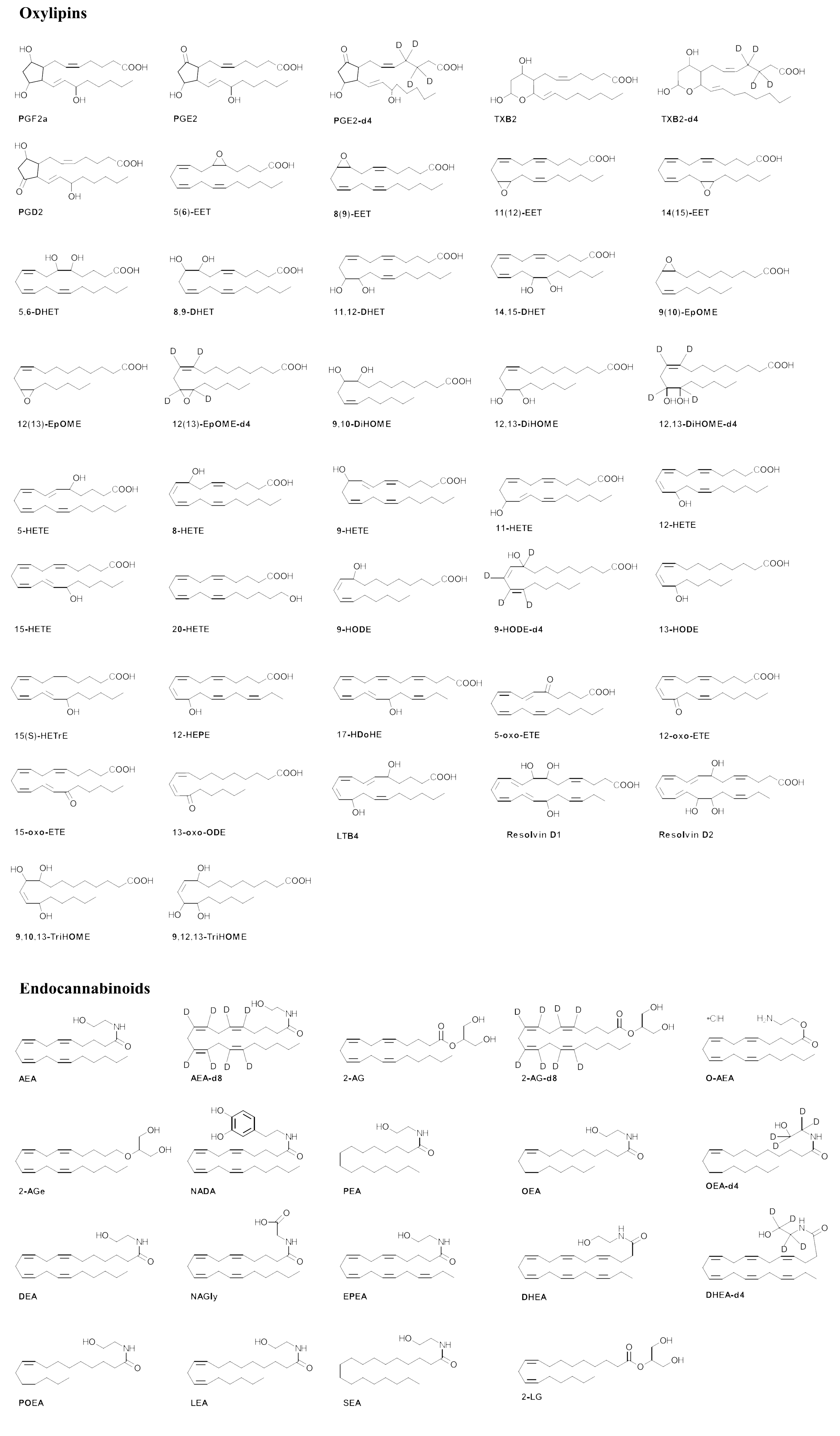

Supplement: S1 Fig — (TIF) [file pone.0132042.s001.tif]

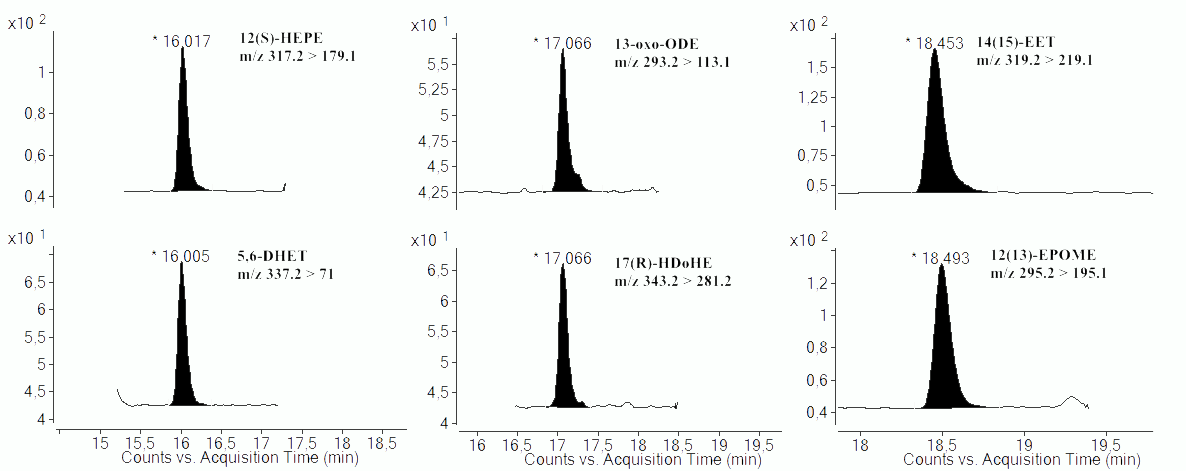

Supplement: S2 Fig — (TIF) [file pone.0132042.s002.tif]

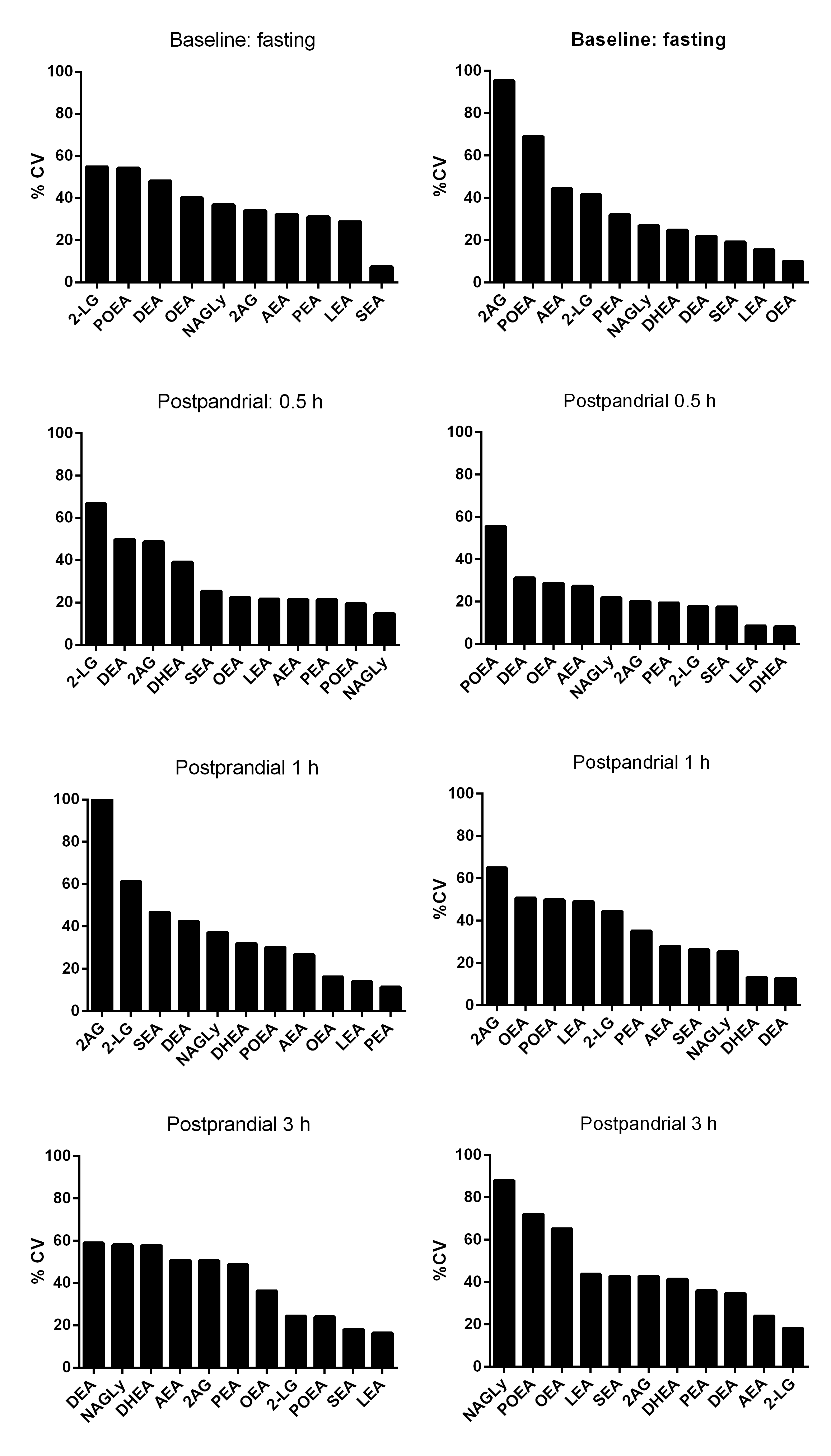

Supplement: S3 Fig — (TIF) [file pone.0132042.s003.tif]

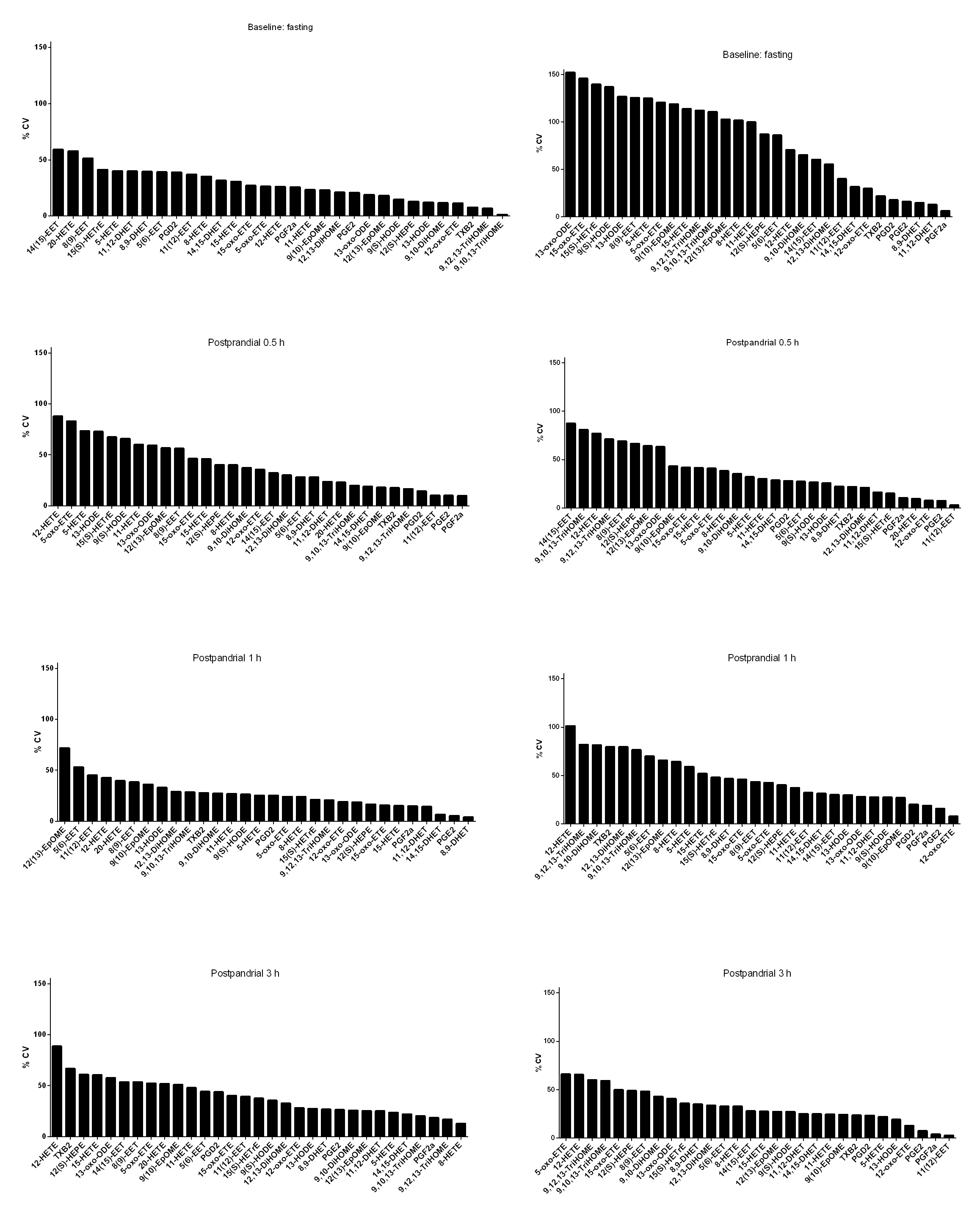

Supplement: S4 Fig — (TIF) [file pone.0132042.s004.tif]

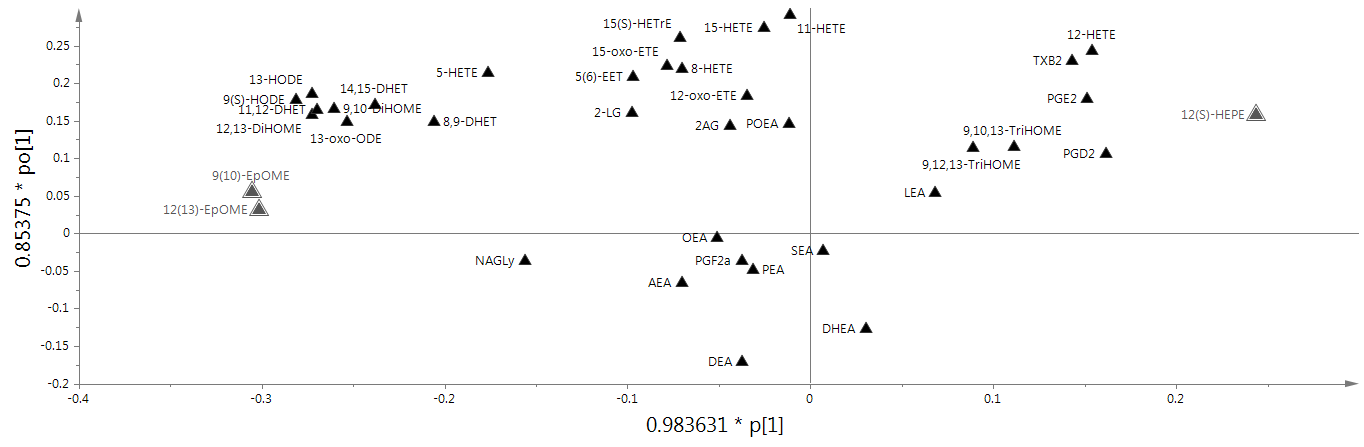

Supplement: S5 Fig — Usual diet-specific metabolites are found to the left (the most typical were 9(10)-EpOME and 12(13)-EpOME, highlighted in grey), and modified diet-specific metabolites are found to the right (the most typical was 12(S)-HEPE, highlighted in grey). (TIF) [file pone.0132042.s005.TIF]
