# Supplementary material for: Profiling the Oxylipin and Endocannabinoid Metabolome by UPLC-ESI-MS/MS in Human Plasma to Monitor Postprandial Inflammation
Source: PLoS One. 2015 Jul 17;10(7):e0132042. doi: 10.1371/journal.pone.0132042 (PMC4506044; doi:10.1371/journal.pone.0132042)
Supplement: S1 Table — P-values below 0.05 are highlighted in bold. (DOCX) [file pone.0132042.s006.docx]

**S1 Table.** Mean values from seven-day dietary records for the subject on vegan (usual diet) and vegetarian (modified) background diets (assessed by two-tailed Student’s *t*-test, if unequal variance, p-value is for t-test assuming unequal variance or Welch's non-parametric test). P-values below 0.05 are highlighted in bold.

|  | **Usual Diet** | | **Modified Diet** | |  |
| --- | --- | --- | --- | --- | --- |
| **Nutrient** | **Mean** | **SD** | **Mean** | **SD** | **p-value** |
| Energy (kcal) | 2392.57 | 659.12 | 2268.00 | 521.35 | 0.70 |
| Protein (g) | 64.33 | 27.20 | 75.04 | 26.43 | 0.47 |
| Total fat (g) | 91.90 | 39.85 | 87.72 | 32.12 | 0.83 |
| Carbohydrate (g) | 338.51 | 87.14 | 298.23 | 74.69 | 0.37 |
| Fiber, total dietary (g) | 38.94 | 8.44 | 28.69 | 6.32 | **0.02** |
| Sugars, total (g) | 111.04 | 25.39 | 100.99 | 32.99 | 0.54 |
| Sucrose (g) | 9.45 | 9.31 | 9.14 | 8.01 | 0.95 |
| Glucose (dextrose) (g) | 8.59 | 4.11 | 6.97 | 3.20 | 0.43 |
| Fructose (g) | 10.84 | 5.97 | 8.29 | 6.70 | 0.47 |
| Starch (g) | 31.43 | 16.24 | 31.72 | 23.78 | 0.98 |
| Calcium, Ca (mg) | 1118.86 | 369.43 | 1733.00 | 568.34 | **0.03** |
| Iron, Fe (mg) | 32.43 | 9.86 | 23.70 | 9.85 | 0.12 |
| Magnesium, Mg (mg) | 549.57 | 261.07 | 287.43 | 126.41 | **0.03** |
| Phosphorus, P (mg) | 1240.43 | 665.98 | 1190.29 | 459.23 | 0.87 |
| Potassium, K (mg) | 2819.29 | 1281.69 | 2249.00 | 459.51 | 0.29 |
| Sodium, Na (mg) | 1963.29 | 635.51 | 2054.86 | 611.36 | 0.79 |
| Zinc, Zn (mg) | 10.11 | 4.06 | 9.55 | 4.07 | 0.80 |
| Copper, Cu (mg) | 2.61 | 1.07 | 1.12 | 0.36 | **0.01** |
| Manganese, Mn (mg) | 6.34 | 2.10 | 5.06 | 2.68 | 0.34 |
| Selenium, Se (mcg) | 53.70 | 13.22 | 64.66 | 19.89 | 0.25 |
| Fluoride, F (mcg) | 899.47 | 615.01 | 658.71 | 434.17 | 0.41 |
| Vitamin C, total ascorbic acid (mg) | 63.01 | 23.99 | 29.07 | 7.86 | **0.01** |
| Thiamin (mg) | 2.14 | 0.90 | 1.45 | 0.51 | 0.10 |
| Riboflavin (mg) | 2.53 | 1.60 | 1.82 | 0.53 | 0.29 |
| Niacin (mg) | 19.56 | 8.65 | 14.79 | 4.94 | 0.23 |
| Pantothenic acid (mg) | 3.13 | 0.60 | 3.96 | 1.32 | 0.16 |
| Vitamin B-6 (mg) | 2.16 | 0.85 | 1.68 | 0.64 | 0.25 |
| Folate, total (mcg) | 528.57 | 303.68 | 321.00 | 116.75 | 0.12 |
| Choline, total (mg) | 141.94 | 72.35 | 92.66 | 41.68 | 0.14 |
| Betaine (mg) | 23.93 | 48.62 | 2.74 | 1.04 | 0.27 |
| Vitamin B-12 (mcg) | 0.51 | 0.75 | 3.28 | 1.66 | **0.002** |
| Vitamin A, IU (IU) | 17362.71 | 15343.22 | 12708.29 | 6735.82 | 0.48 |
| Retinol (mcg) | 519.00 | 116.49 | 788.57 | 306.20 | 0.06 |
| Vitamin E (alpha-tocopherol) (mg) | 6.51 | 3.51 | 7.88 | 5.45 | 0.59 |
| Vitamin D (IU) | 125.54 | 83.29 | 106.77 | 39.94 | 0.60 |
| Vitamin K (phylloquinone) (mcg) | 163.19 | 263.74 | 37.89 | 23.23 | 0.23 |
| Fatty acids, total saturated (g) | 17.99 | 7.40 | 37.85 | 15.32 | **0.01** |
| 4;0 (g) | 0.01 | 0.03 | 1.28 | 0.69 | **0.003** |
| 6;0 (g) | 0.02 | 0.04 | 0.65 | 0.31 | **0.002** |
| 8;0 (g) | 0.04 | 0.09 | 0.46 | 0.18 | **0.0001** |
| 10;0 (g) | 0.04 | 0.08 | 0.99 | 0.26 | **0.00004** |
| 12;0` (g) | 0.24 | 0.58 | 1.19 | 0.55 | **0.01** |
| 14;0 (g) | 0.20 | 0.25 | 3.67 | 1.79 | **0.002** |
| 15;0 (g) | 0.00 | 0.00 | 0.00 | 0.00 |  |
| 16;0 (g) | 8.10 | 3.71 | 13.55 | 5.58 | **0.05** |
| 17;0 (g) | 0.02 | 0.02 | 0.05 | 0.06 | 0.18 |
| 18;0 (g) | 4.06 | 2.13 | 6.11 | 3.20 | 0.18 |
| Fatty acids, total monounsaturated (g) | 27.24 | 15.16 | 25.57 | 11.27 | 0.82 |
| 14;1 (g) | 0.00 | 0.00 | 0.00 | 0.00 | 0.34 |
| 15;1 (g) | 0.00 | 0.00 | 0.00 | 0.00 |  |
| 16;1 undifferentiated (g) | 0.10 | 0.07 | 1.12 | 0.66 | **0.01** |
| 16;1c (g) | 0.02 | 0.04 | 0.08 | 0.08 | 0.14 |
| 17;1 (g) | 0.00 | 0.00 | 0.00 | 0.01 | 0.18 |
| 18:1 undifferentiated (g) | 23.58 | 14.37 | 22.29 | 10.72 | 0.85 |
| 18:1 c (g) | 7.58 | 10.03 | 2.19 | 1.50 | 0.19 |
| 18:1 t (g) | 0.18 | 0.47 | 0.24 | 0.26 | 0.78 |
| 20;1 (g) | 0.11 | 0.07 | 0.09 | 0.06 | 0.51 |
| Fatty acids, total polyunsaturated (g) | 38.19 | 17.25 | 13.70 | 6.69 | **0.01** |
| 18:2 undifferentiated (g) | 31.04 | 15.42 | 10.86 | 5.99 | **0.01** |
| 18:2 n-6 c,c (g) | 11.80 | 15.78 | 1.16 | 0.88 | 0.10 |
| 18:3 undifferentiated (g) | 3.19 | 2.75 | 1.09 | 0.47 | 0.07 |
| 18:3 n-3 c,c,c (g) | 0.17 | 0.23 | 0.07 | 0.07 | 0.27 |
| 20:4 undifferentiated (g) | 0.02 | 0.06 | 0.03 | 0.04 | 0.92 |
| 20:5 n-3 (g) | 0.00 | 0.00 | 0.00 | 0.00 |  |
| 22:6 n-3 (g) | 0.00 | 0.00 | 0.01 | 0.01 | 0.06 |
| Fatty acids, total trans (g) | 0.27 | 0.67 | 0.27 | 0.28 | 0.99 |
| Cholesterol (mg) | 7.14 | 18.90 | 220.00 | 81.05 | **0.0003** |
| Phytosterols (mg) | 179.43 | 135.31 | 92.14 | 73.40 | 0.16 |
| Tryptophan (g) | 0.69 | 0.45 | 0.69 | 0.28 | 1.00 |
| Threonine (g) | 1.98 | 1.28 | 1.89 | 0.67 | 0.87 |
| Isoleucine (g) | 2.16 | 1.40 | 2.43 | 0.99 | 0.69 |
| Leucine (g) | 4.03 | 2.23 | 4.49 | 1.94 | 0.69 |
| Lysine (g) | 2.48 | 2.00 | 3.56 | 1.64 | 0.29 |
| Methionine (g) | 0.84 | 0.42 | 1.16 | 0.47 | 0.20 |
| Cystine (g) | 0.86 | 0.43 | 0.79 | 0.29 | 0.74 |
| Phenylalanine (g) | 2.51 | 1.49 | 2.70 | 1.14 | 0.79 |
| Tyrosine (g) | 1.74 | 1.08 | 2.28 | 1.07 | 0.37 |
| Valine (g) | 2.55 | 1.45 | 3.26 | 1.39 | 0.37 |
| Arginine (g) | 4.59 | 3.04 | 2.63 | 1.02 | 0.13 |
| Histidine (g) | 1.28 | 0.79 | 1.58 | 0.71 | 0.48 |
| Alanine (g) | 2.50 | 1.35 | 2.07 | 0.71 | 0.47 |
| Aspartic acid (g) | 5.30 | 3.80 | 4.21 | 1.26 | 0.48 |
| Glutamic acid (g) | 10.14 | 5.55 | 10.30 | 4.10 | 0.95 |
| Glycine (g) | 2.41 | 1.39 | 1.63 | 0.65 | 0.21 |
| Proline (g) | 2.68 | 1.56 | 4.53 | 2.21 | 0.10 |
| Serine (g) | 2.64 | 1.70 | 2.79 | 1.04 | 0.85 |
| Alcohol, ethyl (g) | 0.00 | 0.00 | 0.00 | 0.00 |  |
| Caffeine (mg) | 50.29 | 43.55 | 40.86 | 31.00 | 0.65 |
| Carotene, beta (mcg) | 7555.86 | 7715.31 | 4571.57 | 3858.18 | 0.38 |
