# Supplementary material for: Profiling the Oxylipin and Endocannabinoid Metabolome by UPLC-ESI-MS/MS in Human Plasma to Monitor Postprandial Inflammation
Source: PLoS One. 2015 Jul 17;10(7):e0132042. doi: 10.1371/journal.pone.0132042 (PMC4506044; doi:10.1371/journal.pone.0132042)
Supplement: S2 Table — (DOCX) [file pone.0132042.s007.docx]

**S2 Table.** Internal standards used for quantification.

| **Internal standard** | **Related native standard** | |
| --- | --- | --- |
| *Endocannabinoids* |  | |
| 2-AG-d_8_ | 2-AG; 2AGe, 2-LG; NADA |  |
| AEA-d_4_ | AEA, *O*-AEA | |
| OEA-d_4_ | PEA, OEA, EPEA, NAGly, POEA, DHEA, LEA, DEA, SEA | |
| *Oxylipins* |  | |
| TXB_2_-d_4_ | TXB_2_, 9,12,13-TriHOME, 9,10,13-TriHOME, | |
| 12(13)-DIHOME-d_4_ | 12(13)-DiHOME, 9(10)-DiHOME, 14,15-DHET, 11,12-DHET, 8,9-DHET, 5,6-DHET, 12(S)-HEPE, 20-HETE | |
| 12(13)-EPOME-d_4_ | 12(13)-EPOME, 9(10)-EPOME | |
| 9(S)-HODE-d_4_ | 13-HODE, 9(S)-HODE, 15-HETE, 17(R)-HDoHE, 13-oxo-ODE, 11-HETE, 15-oxo-ETE, 9-oxo-ODE, 12-HETE, 8-HETE, 15(S)-HETrE, 12-oxo-ETE, 9-HETE, 5-HETE, 14(15)-EET, 11(12)-EET, 8(9)-EET, 5(6)-EET, 5-oxo-ETE | |
| PGE_2_-d_4_ | PGF_2α_, PGE_2_, PGD_2_, Resolvin D1, Resolvin D2, LTB_4_ | |
