# Supplementary material for: Profiling the Oxylipin and Endocannabinoid Metabolome by UPLC-ESI-MS/MS in Human Plasma to Monitor Postprandial Inflammation
Source: PLoS One. 2015 Jul 17;10(7):e0132042. doi: 10.1371/journal.pone.0132042 (PMC4506044; doi:10.1371/journal.pone.0132042)
Supplement: S3 Table — (DOCX) [file pone.0132042.s008.docx]

**S3 Table.** Calibration standard concentrations (µg/mL) for endocannabinoids (stock solution 250 µg/mL).

|  | **Standard concentration (µg/mL)** |
| --- | --- |
| **S1** | 17 |
| **S2** | 3.3 |
| **S3** | 0.67 |
| **S4** | 0.13 |
| **S5** | 0.067 |
| **S6** | 0.033 |
| **S7** | 0.017 |
| **S8** | 0.0083 |
| **S9** | 0.0042 |
| **S10** | 0.00021 |
