# Supplementary material for: Profiling the Oxylipin and Endocannabinoid Metabolome by UPLC-ESI-MS/MS in Human Plasma to Monitor Postprandial Inflammation
Source: PLoS One. 2015 Jul 17;10(7):e0132042. doi: 10.1371/journal.pone.0132042 (PMC4506044; doi:10.1371/journal.pone.0132042)
Supplement: S4 Table — (DOCX) [file pone.0132042.s009.docx]

**S4 Table.** Calibration standard concentrations (pg/mL) for oxylipins.

|  | **Standard concentration (pg/mL)** |
| --- | --- |
| **S1** | 16450 |
| **S2** | 8220 |
| **S3** | 4110 |
| **S4** | 2060 |
| **S5** | 1030 |
| **S6** | 514 |
| **S7** | 257 |
| **S8** | 129 |
| **S9** | 64 |
| **S10** | 37 |
