# Supplementary material for: Profiling the Oxylipin and Endocannabinoid Metabolome by UPLC-ESI-MS/MS in Human Plasma to Monitor Postprandial Inflammation
Source: PLoS One. 2015 Jul 17;10(7):e0132042. doi: 10.1371/journal.pone.0132042 (PMC4506044; doi:10.1371/journal.pone.0132042)
Supplement: S6 Table — (DOCX) [file pone.0132042.s011.docx]

**S6 Table.** Comparison of limit of quantification (LOQ) values with Yang et al [44] and Wang et al [46].

| **Standard** | **LOQ (pg on column)** | **LOQ (pg on column)**  Ref #34 (Yang *et al* 2009, *Anal Chem.* 81:8085) | **LOQ (pg on column)**  Ref # 46 (Wang *et al* 2014, *J. Chromatog. A*, 1359: 60) |
| --- | --- | --- | --- |
| TXB_2_ | 0.05 | 0.74 | 3 |
| 9,12,13-TriHOME | 0.01 | 0.07 | - |
| 9,10,13-TriHOME | 0.05 | 0.07 | - |
| PGF_2α_ | 0.05 | 0.35 | 70 |
| PGE_2_ | 0.05 | 0.07 | 3 |
| PGD_2_ | 0.1 | 0.70 | 3 |
| Resolvin D2 | 1.05 | - | - |
| Resolvin D1 | 0.26 | - | 5 |
| LTB4 | 0.26 | 0.20 | 3 |
| 12(13)-DiHOME | 0.05 | 0.63 | 3 |
| 9(10)-DiHOME | 0.005 | 0.63 | 3 |
| 14,15-DHET | 0.05 | 0.07 | 3 |
| 11,12-DHET | 0.26 | 0.20 | 3 |
| 8,9-DHET | 0.26 | 0.67 | 3 |
| 5,6-DHET | 0.26 | 0.67 | 3 |
| 12(S)-HEPE | 0.05 | - | 3 |
| 20-HETE | 1.05 | 15.9 | 3 |
| 13-HODE | 0.26 | 0.30 | 3 |
| 9(S)-HODE | 0.005 | 0.30 | 3 |
| 15-HETE | 0.05 | 0.32 | 3 |
| 17(R)-HDoHE | 1.05 | - | 30 |
| 13-oxo-ODE | 1.05 | 14.6 | 10 |
| 11-HETE | 0.01 | 0.96 | 1 |
| 15-oxo-ETE | 0.26 | 0.03 | 3 |
| 9-oxo-ODE | 135 | 0.88 | 3 |
| 12-HETE | 0.26 | 0.32 | 3 |
| 8-HETE | 0.26 | 3.19 | 5 |
| 15(S)-HETrE | 0.01 | - | 3 |
| 12-oxo-ETE | 0.05 | - | 5 |
| 9-HETE | 4.21 | 0.32 | 3 |
| 5-HETE | 0.26 | 0.32 | 3 |
| 12(13)-EpOME | 0.53 | 0.59 | 3 |
| 14(15)-EET | 0.05 | 0.96 | 3 |
| 9(10)-EpOME | 0.53 | 0.06 | 3 |
| 11(12)-EET | 0.05 | 0.32 | 3 |
| 5-oxo-ETE | 0.26 | 15.8 | 3 |
| 8(9)-EET | 0.26 | 0.96 | 7 |
| 5(6)-EET | 0.53 | 3.19 | 3 |
