# Supplementary material for: Profiling the Oxylipin and Endocannabinoid Metabolome by UPLC-ESI-MS/MS in Human Plasma to Monitor Postprandial Inflammation
Source: PLoS One. 2015 Jul 17;10(7):e0132042. doi: 10.1371/journal.pone.0132042 (PMC4506044; doi:10.1371/journal.pone.0132042)
Supplement: S7 Table — (DOCX) [file pone.0132042.s012.docx]

**S7 Table.** Inter- and intraday accuracy and precision (coefficient of variation) for quality control (QC) samples at four different concentration levels.

| **Compound** | **QC 1 (96.0 pg/µL)** | | | | **QC 2 (23.9 pg/µL)** | | | | **QC 3 (12.0 pg/µL)** | | | | **QC 4 (2.99 pg/µL)** | | | |
| --- | --- | --- | --- | --- | --- | --- | --- | --- | --- | --- | --- | --- | --- | --- | --- | --- |
|  | **Intraday** | | **Interday** | | **Intraday** | | **Interday** | | **Intraday** | | **Interday** | | **Intraday** | | **Interday** | |
|  | Acc. [%] | Prec. [%] | Acc. [%] | Prec.[%] | Acc. [%] | Prec.[%] | Acc. [%] | Prec.[%] | Acc. [%] | Prec.[%] | Acc. [%] | Prec.[%] | Acc. [%] | Prec. [%] | Acc. [%] | Prec.[%] |
| TXB_2_ | 90.3 | 0.62 | 98.6 | 1.53 | 88.5 | 3.31 | 97.3 | 4.80 | 89.2 | 2.19 | 96.6 | 2.59 | 93.3 | 2.82 | 109 | 5.80 |
| 9,12,13-TriHOME | 115 | 2.10 | 121 | 2.45 | 104 | 3.38 | 116 | 3.93 | 105 | 1.37 | 112 | 2.24 | 105 | 1.71 | 108 | 4.65 |
| 9,10,13-TriHOME | 108 | 1.26 | 113 | 3.76 | 120 | 5.21 | 117 | 4.53 | 102 | 3.94 | 106 | 3.19 | 48.1 | 7.90 | 51.3 | 11.2 |
| PGF_2α_ | 104 | 3.73 | 113 | 1.96 | 128 | 5.87 | 121 | 5.75 | 119 | 1.80 | 114 | 5.71 | 135 | 1.50 | 175 | 3.49 |
| PGE_2_ | 101 | 3.73 | 104 | 3.18 | 101 | 5.87 | 100 | 3.31 | 98.2 | 1.80 | 99.9 | 3.25 | 113 | 1.50 | 107 | 2.43 |
| PGD_2_ | 98 | 4.27 | 94.5 | 3.00 | 105 | 1.12 | 100 | 2.08 | 108 | 2.63 | 99.2 | 3.53 | 110 | 1.54 | 102 | 2.42 |
| Resolvin D2 | 81.1 | 1.42 | 80.1 | 4.54 | 86.9 | 10.5 | 86.1 | 13.9 | 81.5 | 9.20 | 81.1 | 12.6 | 80.6 | 9.08 | 85.2 | 8.59 |
| Resolvin D1 | 101 | 3.27 | 103 | 3.30 | 102 | 4.40 | 103 | 2.26 | 102 | 0.86 | 99.7 | 2.42 | 97.7 | 4.87 | 100 | 4.14 |
| LTB_4_ | 105 | 3.46 | 111 | 2.16 | 100 | 1.96 | 115 | 2.12 | 101 | 2.50 | 111 | 3.48 | 106 | 3.22 | 112 | 2.47 |
| 12(13)-DiHOME | 105 | 2.82 | 105 | 2.71 | 112 | 1.69 | 112 | 2.03 | 114 | 3.27 | 113 | 2.05 | 119 | 1.20 | 120 | 1.77 |
| 9(10)-DiHOME | 107 | 3.40 | 102 | 2.50 | 114 | 3.16 | 114 | 2.31 | 116 | 4.08 | 116 | 4.30 | 113 | 0.50 | 113 | 2.66 |
| 14,15-DHET | 106 | 3.03 | 105 | 2.74 | 108 | 1.44 | 108 | 2.05 | 106 | 1.52 | 107 | 3.03 | 106 | 3.63 | 105 | 3.12 |
| 11,12-DHET | 106 | 1.08 | 104 | 1.94 | 108 | 3.60 | 107 | 2.49 | 108 | 1.38 | 106 | 3.05 | 109 | 1.09 | 108 | 3.15 |
| 8,9-DHET | 107 | 3.07 | 94.2 | 3.04 | 102 | 3.01 | 101 | 2.33 | 112 | 5.34 | 110 | 4.30 | 107 | 2.26 | 111 | 2.14 |
| 5,6-DHET | 106 | 0.82 | 105 | 2.22 | 102 | 2.99 | 110 | 2.48 | 108 | 8.04 | 110 | 5.72 | 121 | 5.26 | 113 | 3.67 |
| 12-HEPE | 106 | 1.61 | 105 | 3.21 | 114 | 2.19 | 113 | 2.15 | 111 | 2.77 | 109 | 3.06 | 118 | 3.18 | 113 | 3.46 |
| 20-HETE | 104 | 2.28 | 104 | 2.72 | 108 | 3.34 | 108 | 2.22 | 109 | 0.65 | 107 | 1.88 | 116 | 1.92 | 109 | 3.96 |
| 13-HODE | 89.9 | 3.03 | 100 | 3.59 | 89.3 | 4.76 | 101 | 4.09 | 93.4 | 4.68 | 99.6 | 3.15 | 100 | 4.18 | 112 | 5.11 |
| 9-HODE | 86.4 | 4.29 | 99.9 | 3.09 | 89.5 | 2.34 | 105 | 5.02 | 91.55 | 2.12 | 101 | 4.05 | 96.5 | 8.19 | 108 | 6.65 |
| 15-HETE | 91.9 | 0.55 | 104 | 2.68 | 87.5 | 3.80 | 103 | 5.98 | 90.5 | 4.43 | 97.1 | 3.87 | 112 | 6.53 | 105 | 5.91 |
| 17(R)-HDoHE | 92.8 | 3.48 | 101 | 2.62 | 81.1 | 1.87 | 93.9 | 7.36 | 86.9 | 4.43 | 91.1 | 4.23 | 105 | 8.87 | 96.3 | 4.30 |
| 13-oxo-ODE | 108 | 3.89 | 101 | 3.18 | 113 | 3.36 | 107 | 4.71 | 108 | 10.3 | 107 | 7.92 | 104 | 14.52 | 113 | 11.0 |
| 11-HETE | 87.2 | 2.81 | 102 | 2.28 | 85.9 | 2.11 | 102 | 5.58 | 87.5 | 7.07 | 97.9 | 6.15 | 104 | 5.23 | 106 | 6.35 |
| 15-oxo-ETE | 109 | 2.38 | 108 | 2.35 | 112 | 0.79 | 111 | 4.47 | 107 | 2.58 | 110 | 4.23 | 110 | 5.75 | 117 | 5.18 |
| 12-HETE | 98.1 | 3.42 | 107 | 2.76 | 94.7 | 3.36 | 109 | 4.75 | 93.1 | 5.75 | 103 | 5.04 | 118 | 6.32 | 113 | 7.62 |
| 8-HETE | 92.47 | 2.34 | 107 | 2.78 | 89.5 | 3.74 | 109 | 4.91 | 93.8 | 6.78 | 104 | 3.52 | 104 | 10.92 | 107 | 7.00 |
| 15(S)-HETrE | 95.1 | 5.46 | 103 | 3.62 | 95.4 | 6.50 | 105 | 6.70 | 96.9 | 0.11 | 100 | 2.54 | 116 | 7.72 | 107 | 7.04 |
| 12-oxo-ETE | 115 | 2.62 | 121 | 3.51 | 118 | 5.49 | 129 | 8.54 | 127 | 8.68 | 127 | 6.15 | 134 | 17.39 | 129 | 10.5 |
| 9-HETE | 91.2 | 1.46 | 105 | 2.46 | 93.9 | 4.38 | 107 | 6.97 | 99.7 | 4.90 | 104 | 3.99 | 109 | 5.66 | 107 | 10.4 |
| 5-HETE | 89.2 | 2.49 | 102 | 2.49 | 88.7 | 2.71 | 101 | 4.65 | 92.6 | 6.04 | 101 | 6.00 | 91.6 | 12.24 | 100 | 8.92 |
| 12(13)-EpOME | 110 | 8.57 | 122 | 7.68 | 121 | 5.85 | 135 | 5.79 | 126 | 2.18 | 149 | 5.77 | 144 | 8.98 | 152 | 8.04 |
| 14(15)-EET | 97.7 | 2.43 | 103 | 3.32 | 98.1 | 11.7 | 107 | 9.31 | 106 | 4.07 | 105 | 8.56 | 108 | 6.30 | 105 | 11.1 |
| 9(10)-EpOME | 104 | 7.99 | 116 | 6.72 | 106 | 7.50 | 119 | 4.54 | 110 | 2.84 | 123 | 6.90 | 114 | 13.12 | 119 | 9.26 |
| 11(12)-EET | 106 | 3.74 | 101 | 2.62 | 104 | 1.52 | 119 | 5.55 | 107 | 6.37 | 99.8 | 5.10 | 98.6 | 14.40 | 114 | 9.21 |
| 5-oxo-ETE | 101 | 1.23 | 108 | 1.49 | 100 | 8.56 | 107 | 5.04 | 108 | 2.37 | 105 | 6.93 | 103 | 9.09 | 102 | 10.2 |
| 8(9)-EET | 108 | 2.07 | 107 | 2.29 | 108 | 4.47 | 114 | 9.39 | 116 | 4.28 | 109 | 4.40 | 115 | 10.77 | 108 | 6.09 |
| 5(6)-EET | 92.6 | 9.26 | 107 | 5.33 | 93.4 | 10.7 | 114 | 8.60 | 126 | 0.68 | 132 | 4.42 | 140 | 5.76 | 157 | 6.12 |
