# Supplementary material for: Profiling the Oxylipin and Endocannabinoid Metabolome by UPLC-ESI-MS/MS in Human Plasma to Monitor Postprandial Inflammation
Source: PLoS One. 2015 Jul 17;10(7):e0132042. doi: 10.1371/journal.pone.0132042 (PMC4506044; doi:10.1371/journal.pone.0132042)
Supplement: S8 Table — (DOCX) [file pone.0132042.s013.docx]

**S8 Table**. Levels (nM) and stability of 5 pooled human plasma samples (expressed as % of the 0 day value) for the metabolites (oxylipins, endocannabinoids and related compounds) under study.

|  | Day 0 | Day 7 |  | Day 31 |  | Day 300 | | Two freeze-thaw cycles at -20°C | | |  |
| --- | --- | --- | --- | --- | --- | --- | --- | --- | --- | --- | --- |
|  | Conc (nM) | Conc (nM) | Stability (%) | Conc (nM) | Stability (%) | Conc nM | Stability (%) | Conc (nM) | Stability (%) |  |  |
| *Oxylipins* |  |  |  |  |  |  |  |  |  |  |  |
| TXB_2_ | 2.88 | 2.92 | 101.3 | 2.72 | 94.4 | 2.65 | 92.0 | 2.74 | 95.1 |  |  |
| 9,12,13-TriHOME | 2.52 | 2.45 | 97.1 | 2.50 | 99.1 | 2.44 | 96.8 | 2.31 | 91.7 |  |  |
| 9,10,13-TriHOME | 0.48 | 0.50 | 104.5 | 0.47 | 97.8 | 0.46 | 94.7 | 0.35 | 71.8 |  |  |
| PGF_2α_ | 0.10 | 0.09 | 92.9 | 0.08 | 82.5 | 0.07 | 76.2 | 0.07 | 74.5 |  |  |
| PGE_2_ | 0.15 | 0.13 | 90.6 | 0.14 | 96.0 | 0.14 | 94.2 | 0.12 | 82.3 |  |  |
| PGD_2_ | 0.07 | 0.07 | 93.7 | 0.07 | 94.3 | 0.06 | 89.9 | 0.07 | 90.4 |  |  |
| 12(13)-DiHOME | 4.38 | 4.56 | 104.0 | 4.45 | 101.5 | 4.20 | 95.8 | 4.16 | 94.9 |  |  |
| 9(10)-DiHOME | 2.49 | 2.35 | 94.6 | 2.32 | 93.3 | 2.29 | 92.1 | 2.02 | 81.4 |  |  |
| 14.15-DHET | 0.44 | 0.45 | 101.6 | 0.42 | 94.4 | 0.40 | 89.9 | 0.39 | 88.3 |  |  |
| 11.12-DHET | 0.16 | 0.15 | 97.5 | 0.15 | 94.7 | 0.14 | 88.4 | 0.12 | 74.6 |  |  |
| 8.9-DHET | 0.10 | 0.09 | 90.9 | 0.09 | 87.9 | 0.08 | 79.0 | 0.06 | 59.8 |  |  |
| 5.6-DHET | 0.08 | 0.08 | 97.3 | 0.08 | 95.2 | 0.07 | 93.1 | 0.06 | 76.5 |  |  |
| 12-HEPE | 0.40 | 0.42 | 105.1 | 0.39 | 96.7 | 0.37 | 92.0 | 0.34 | 84.5 |  | |
| 20-HETE | 0.17 | 0.17 | 102.7 | 0.17 | 98.5 | 0.14 | 83.7 | 0.13 | 77.6 |  | |
| 13-HODE | 7.48 | 7.50 | 100.3 | 7.32 | 97.9 | 7.20 | 96.3 | 7.04 | 94.1 |  | |
| 9-HODE | 5.55 | 5.56 | 100.1 | 5.40 | 97.3 | 5.19 | 93.6 | 4.94 | 89.0 |  | |
| 15-HETE | 0.39 | 0.37 | 94.1 | 0.36 | 91.6 | 0.35 | 88.9 | 0.28 | 70.6 |  | |
| 17(R)-HDoHE | 0.53 | 0.53 | 100.1 | 0.52 | 98.7 | 0.51 | 95.7 | 0.48 | 91.1 |  | |
| 13-oxo-ODE | 0.57 | 0.54 | 95.9 | 0.54 | 94.7 | 0.50 | 88.9 | 0.50 | 88.2 |  | |
| 15-oxo-ETE | 0.04 | 0.03 | 82.8 | 0.03 | 77.4 | 0.02 | 40.6 | 0.01 | 32.8 |  | |
| 11-HETE | 0.16 | 0.18 | 112.6 | 0.16 | 95.7 | 0.13 | 82.3 | 0.13 | 77.7 |  | |
| 12-HETE | 7.17 | 7.20 | 100.4 | 7.18 | 100.1 | 6.72 | 93.7 | 6.53 | 91.0 |  | |
| 8-HETE | 0.13 | 0.13 | 103.3 | 0.12 | 92.6 | 0.11 | 85.0 | 0.09 | 68.4 |  | |
| 15(S)-HETrE | 0.11 | 0.11 | 99.2 | 0.11 | 99.2 | 0.09 | 81.3 | 0.08 | 73.0 |  | |
| 12-oxo-ETE | 0.25 | 0.24 | 96.4 | 0.24 | 95.2 | 0.24 | 95.6 | 0.22 | 87.9 |  | |
| 5-HETE | 0.15 | 0.15 | 99.9 | 0.15 | 96.2 | 0.13 | 85.8 | 0.19 | 125.4 |  | |
| 12(13)-EpOME | 0.28 | 0.30 | 106.1 | 0.29 | 101.8 | 0.27 | 97.0 | 0.22 | 79.7 |  | |
| 9(10)-EpOME | 0.44 | 0.43 | 99.3 | 0.42 | 95.4 | 0.39 | 89.1 | 0.36 | 82.0 |  | |
| 11(12)-EET | 0.09 | 0.10 | 106.4 | 0.09 | 97.0 | 0.07 | 78.7 | 0.07 | 74.0 |  | |
| 8(9)-EET | 0.08 | 0.08 | 101.6 | 0.08 | 98.7 | 0.08 | 95.3 | 0.06 | 79.3 |  | |
| 5(6)-EET | 0.29 | 0.30 | 104.8 | 0.29 | 101.3 | 0.25 | 87.6 | 0.22 | 76.8 |  | |
| *Endocannabinoids* |  |  |  |  |  |  |  |  |  |  | |
| NAGly | 2.23 | 2.18 | 97.8 | 2.06 | 92.4 | 1.73 | 77.6 | 1.62 | 72.6 |  | |
| EPEA | 0.54 | 0.55 | 101.9 | 0.49 | 90.7 | 0.38 | 70.4 | 0.36 | 66.7 |  | |
| POEA | 1.28 | 1.24 | 96.9 | 1.19 | 93.0 | 1.08 | 84.4 | 1.06 | 82.8 |  | |
| DHEA | 0.03 | 0.03 | 98.1 | 0.03 | 87.1 | 0.02 | 67.7 | 0.02 | 58.1 |  | |
| AEA | 0.49 | 0.48 | 96.9 | 0.43 | 87.8 | 0.40 | 81.6 | 0.39 | 79.6 |  | |
| LEA | 3.32 | 3.30 | 99.4 | 3.15 | 94.9 | 2.98 | 89.8 | 2.83 | 85.2 |  | |
| 2-AG | 15.07 | 14.76 | 97.9 | 12.78 | 84.8 | 11.50 | 76.3 | 10.30 | 68.3 |  | |
| PEA | 8.34 | 8.12 | 97.4 | 7.89 | 94.6 | 7.14 | 85.6 | 7.09 | 85.0 |  | |
| DEA | 4.52 | 4.43 | 98.0 | 4.21 | 93.1 | 3.87 | 85.6 | 3.56 | 78.8 |  | |
| OEA | 3.64 | 3.58 | 98.4 | 3.35 | 92.0 | 3.22 | 88.5 | 3.16 | 86.8 |  | |
| SEA | 5.39 | 5.31 | 98.5 | 4.87 | 90.4 | 4.52 | 83.9 | 4.41 | 81.8 |  | |
