# Supplementary material for: Profiling the Oxylipin and Endocannabinoid Metabolome by UPLC-ESI-MS/MS in Human Plasma to Monitor Postprandial Inflammation
Source: PLoS One. 2015 Jul 17;10(7):e0132042. doi: 10.1371/journal.pone.0132042 (PMC4506044; doi:10.1371/journal.pone.0132042)
Supplement: S9 Table — (DOCX) [file pone.0132042.s014.docx]

**S9 Table.** Oxylipin levels (nM) in the plasma samples from the challenge meal study in the fasting state (0) and postprandial state (at 0.5, 1, 3 hours after the meal) at six different occasions (a – f ).

Usual Diet

Modified Diet
