# Supplementary material for: Profiling the Oxylipin and Endocannabinoid Metabolome by UPLC-ESI-MS/MS in Human Plasma to Monitor Postprandial Inflammation
Source: PLoS One. 2015 Jul 17;10(7):e0132042. doi: 10.1371/journal.pone.0132042 (PMC4506044; doi:10.1371/journal.pone.0132042)
Supplement: S10 Table — (DOCX) [file pone.0132042.s015.docx]

**S10 Table.** Coefficient of variation (CV) values (%) for oxylipins in the fasting state and postprandial state (at 0.5, 1, 3 hours after the meal), and in quality control (QC) samples.

Usual Diet

Modified Diet
