# Supplementary material for: Profiling the Oxylipin and Endocannabinoid Metabolome by UPLC-ESI-MS/MS in Human Plasma to Monitor Postprandial Inflammation
Source: PLoS One. 2015 Jul 17;10(7):e0132042. doi: 10.1371/journal.pone.0132042 (PMC4506044; doi:10.1371/journal.pone.0132042)
Supplement: S11 Table — (DOCX) [file pone.0132042.s016.docx]

**S11Table.** Coefficient of variation (CV) values (%) for endocannabinoids in the fasting state and postprandial state (at 0.5, 1, 3 hours after the meal), and in quality control (QC) samples [32].

|  | | | Usual Diet | | | Modified Diet | |  | | | |  | | QC1 8.10 ng/mL | | | QC2 325 pg/mL | | | QC3 64.9 pg/mL | | | QC4 8.11 pg/mL | |
| --- | --- | --- | --- | --- | --- | --- | --- | --- | --- | --- | --- | --- | --- | --- | --- | --- | --- | --- | --- | --- | --- | --- | --- | --- |
|  | baseline | 0.5 h | | 1 h | 3 h | | baseline | | 0.5 h | 1 h | 3 h | | intraday | | interday | intraday | | interday | intraday | | interday | intraday | | interday |
| 2AG | 34 | 49 | | 135 | 51 | | 95 | | 20 | 65 | 43 | | 7.7 | | 6.8 | 0.8 | | 5.5 | 4.8 | | 5.5 | 7.9 | | 9.5 |
| 2-LG | 55 | 67 | | 61 | 24 | | 41 | | 18 | 44 | 18 | | 7.9 | | 5.3 | 2.9 | | 11 | ND | | ND | ND | | ND |
| AEA | 32 | 21 | | 27 | 51 | | 44 | | 27 | 28 | 24 | | 0.9 | | 8.5 | 6.5 | | 12 | 11 | | 19 | 6.8 | | 8.8 |
| DEA | 48 | 50 | | 42 | 59 | | 22 | | 31 | 13 | 35 | | 2.3 | | 9.5 | 2.5 | | 6.6 | 3.9 | | 8.6 | 9.8 | | 9.5 |
| DHEA |  | 39 | | 32 | 58 | | 25 | | 8 | 13 | 41 | | 4.5 | | 14 | 2.9 | | 6.8 | 1.2 | | 12 | ND | | ND |
| LEA | 29 | 22 | | 14 | 16 | | 15 | | 9 | 49 | 44 | | 1.6 | | 6.8 | 0.9 | | 5.5 | 5.0 | | 4.3 | 2.7 | | 4.9 |
| NAGLy | 37 | 15 | | 37 | 58 | | 27 | | 22 | 25 | 88 | | 1.2 | | 16 | 9.4 | | 13 | 4.2 | | 8.8 | 6.6 | | 14 |
| OEA | 40 | 22 | | 16 | 36 | | 10 | | 29 | 51 | 65 | | 1.6 | | 7.3 | 5.8 | | 4.4 | 7.0 | | 5.0 | 5.5 | | 3.8 |
| PEA | 31 | 21 | | 11 | 49 | | 32 | | 19 | 35 | 36 | | 1.4 | | 4.1 | 2.4 | | 4.8 | 1.3 | | 3.4 | 1.5 | | 10 |
| POEA | 54 | 19 | | 30 | 24 | | 69 | | 56 | 50 | 72 | | 0.87 | | 5.6 | 4.7 | | 7.2 | 1.0 | | 16 | 6.5 | | 11 |
| SEA | 7 | 25 | | 47 | 18 | | 19 | | 18 | 26 | 43 | | 1.4 | | 2.9 | 9.3 | | 7.5 | 5.8 | | 5.9 | 3.5 | | 6.4 |
