# Supplementary material for: Profiling the Oxylipin and Endocannabinoid Metabolome by UPLC-ESI-MS/MS in Human Plasma to Monitor Postprandial Inflammation
Source: PLoS One. 2015 Jul 17;10(7):e0132042. doi: 10.1371/journal.pone.0132042 (PMC4506044; doi:10.1371/journal.pone.0132042)
Supplement: S12 Table — (DOCX) [file pone.0132042.s017.docx]

**S12 Table.** Variable influence on projection (VIP) values for each metabolite in the OPLS-DA model.

| **Metabolite** | **M5.VIP[1]** |
| --- | --- |
| **9(10)-EpOME** | 2.0 |
| **11,12-DHET** | 1.8 |
| **12(13)-EpOME** | 1.7 |
| **9(S)-HODE** | 1.7 |
| **13-oxo-ODE** | 1.6 |
| **12(13)-DiHOME** | 1.6 |
| **13-HODE** | 1.5 |
| **14,15-DHET** | 1.5 |
| **9(10)-DiHOME** | 1.5 |
| **12(S)-HEPE** | 1.3 |
| **8,9-DHET** | 1.3 |
| **PGD_2_** | 1.1 |
| **5-HETE** | 1.0 |
| **9,10,13-TriHOME** | 0.9 |
| **NAGLy** | 0.8 |
| **LEA** | 0.8 |
| **5(6)-EET** | 0.7 |
| **8-HETE** | 0.7 |
| **12-HETE** | 0.7 |
| **15-oxo-ETE** | 0.7 |
| **9,12,13-TriHOME** | 0.7 |
| **PGE_2_** | 0.7 |
| **TXB_2_** | 0.6 |
| **2-LG** | 0.5 |
| **15(S)-HETrE** | 0.5 |
| **15-HETE** | 0.4 |
| **2AG** | 0.4 |
| **AEA** | 0.4 |
| **SEA** | 0.4 |
| **DHEA** | 0.3 |
| **12-oxo-ETE** | 0.3 |
| **DEA** | 0.3 |
| **PGF_2α_** | 0.3 |
| **11-HETE** | 0.2 |
| **PEA** | 0.2 |
| **POEA** | 0.1 |
| **OEA** | 0.1 |
